# Supplementary material for: Engagement of primary care physicians in medication decision-making for patients with multimorbidity in China: A cross-sectional study
Source: PLoS One. 2026 Mar 26;21(3):e0344518. doi: 10.1371/journal.pone.0344518 (PMC13020813; doi:10.1371/journal.pone.0344518)
Supplement: S1 File — (DOCX) [file pone.0344518.s001.docx]

**Supporting 1 File**

**A survey on primary care physicians’ engagement in medication decision-making for patients with multimorbidity**

Dear physician,

Greetings! We are members of the Multimorbidity and Medication Decision-Making research team at Zhejiang University. We are conducting a cross-sectional survey regarding primary care physicians’ engagement in medication decision-making for patients with multimorbidity. The purpose of this survey is to understand your practices and perceived difficulties when making medication decisions for patients with multimorbidity in outpatient settings. By collecting your insights, we aim to provide an important reference for developing future implementation strategies to enhance the quality of medication therapy for multimorbidity in primary care.

Therefore, we sincerely invite you to participate in this survey. The questionnaire is anonymous, and there are no right or wrong answers. Please fill it out based on your actual practices and perspectives, selecting the most appropriate options. Completing this survey will take approximately 3 to 5 minutes. We assure you of the confidentiality of your information; all data will be kept secure and not disclosed. Please note that participation in this survey is entirely voluntary. You are also free to decide whether to participate and may withdraw at any time. We sincerely appreciate your support and participation!

**Informed consent for participation in this survey**

□ I have read the above information and voluntarily agree to participate in the survey.
□ I decline to participate in this survey.

**Part Ⅰ. General Information**

1. Your gender:

□Male □Female

1. Age: ____________ (years)
2. Duration of clinical practice: _____________ (years)
3. Your professional title:

□ Resident physician □ Attending physician

□ Associate chief physician □ Chief physician

1. Your highest academic degree:

□ College

□ Bachelor

□ Master or doctor

1. Have you completed the standardized residency training?

□Yes □No

1. Are you responsible for contracted family doctor services?

□Yes □No

1. Location of your PHC facility:

□Rural □Urban

1. In the past year, have you participated in pharmaceutical training?

□Yes □No

1. In the past month, approximately how many patients did you see daily on average in outpatient clinic? ____________
2. Does the PHC facility regularly conduct prescription reviews?

□Yes □No

1. Is there available CDSS for medication inquiry in your PHC facility?

□Yes □No

1. When facing medication-related problems, do you usually collaborate with pharmacists to resolve these problems?

□Yes □No

**Part Ⅱ:**  **Medication decision-making practices**

*Instructions: For the following questions, please select the appropriate option based on the frequency of your behaviors related to medication decision-making for patients with multimorbidity during outpatient consultations over the past month.*

**Prior to formulating medication therapy decisions, I typically:**

1. Understand the patients’ purpose of the visit.

□ Always □ Occasionally □ Rarely

1. Address patients’ needs to be prioritized.

□ Always □ Occasionally □ Rarely

1. Review patient’s medical history and medication history.

□ Always □ Occasionally □ Rarely

1. Assess the appropriateness and effectiveness of the original therapy.

□ Always □ Occasionally □ Rarely

1. Assess potential drug-drug and/or drug-disease interactions.

□ Always □ Occasionally □ Rarely

1. Assess patient’s medication adherence and treatment burden.

□ Always □ Occasionally □ Rarely

**During the decision-making process, I typically:**

1. Establish mutually agreed treatment goals with the patients.

□ Always □ Occasionally □ Rarely

1. Encourage the patients to participate in the decision-making process.

□ Always □ Occasionally □ Rarely

1. Elicit the patient’s expectations for treatment outcomes.

□ Always □ Occasionally □ Rarely

1. Discuss the benefits and feasibility of medications with the patients.

□ Always □ Occasionally □ Rarely

1. Discuss the potential risks of medications with the patients.

□ Always □ Occasionally □ Rarely

1. Inform patients about how to manage medication side effects.

□ Always □ Occasionally □ Rarely

1. Provide alternatives of treatment plan .

□ Always □ Occasionally □ Rarely

1. Understand patients’ preferences for medication therapy.

□ Always □ Occasionally □ Rarely

**After formulating a medication therapy plan for the patients, I typically:**

1. Document the patient's medication information accurately.

□ Always □ Occasionally □ Rarely

1. Inquire about the patient’s further questions regarding medication use.

□ Always □ Occasionally □ Rarely

1. Provide instructions on how to use the medication.

□ Always □ Occasionally □ Rarely

1. Provide instructions on relevant precautions for medication use.

□ Always □ Occasionally □ Rarely

1. Inform about the common manifestations of adverse drug reactions.

□ Always □ Occasionally □ Rarely

1. Inform the patient about the follow-up appointment.

□ Always □ Occasionally □ Rarely

**Part III. Perceived difficulties in decision-making**

*Instructions: I find the occurrence of the situations described in the following statements difficult and challenging when making medication decisions for patients with multimorbidity.*

1. Determine that all medications have indications.

◻ Strongly agree ◻ Agree ◻ Disagree ◻ Strongly disagree

1. Identify drug-drug/ disease interactions.

◻ Strongly agree ◻ Agree ◻ Disagree ◻ Strongly disagree

1. Explain potential adverse outcomes of medication therapy.

◻ Strongly agree ◻ Agree ◻ Disagree ◻ Strongly disagree

1. Understand the correct usage of multiple medications.

◻ Strongly agree ◻ Agree ◻ Disagree ◻ Strongly disagree

1. Balance the treatment benefits against risks.

◻ Strongly agree ◻ Agree ◻ Disagree ◻ Strongly disagree

1. Unable to make new decisions due to clinical inertia.

◻ Strongly agree ◻ Agree ◻ Disagree ◻ Strongly disagree

1. Discuss the alternative treatment options with patients.

◻ Strongly agree ◻ Agree ◻ Disagree ◻ Strongly disagree

1. Handle the potential negative consequences.

◻ Strongly agree ◻ Agree ◻ Disagree ◻ Strongly disagree
