# Supplementary material for: Engagement of primary care physicians in medication decision-making for patients with multimorbidity in China: A cross-sectional study
Source: PLoS One. 2026 Mar 26;21(3):e0344518. doi: 10.1371/journal.pone.0344518 (PMC13020813; doi:10.1371/journal.pone.0344518)
Supplement: S2 File — (PDF) [file pone.0344518.s002.pdf]

ID	Completed t	Family doctor c	Number of outp	Received	pharmaceutical	The facility regu	Available CDSS	Collaborate with	Understand the	Address the pat
1	No	Yes	30		Yes	Yes	No	No	always	occasionally
2	No	Yes	10		No	No	No	Yes	occasionally	occasionally
3	No	Yes	25		No	Yes	Yes	No	always	always
4	No	Yes	60		Yes	Yes	Yes	Yes	always	always
5	Yes	No	60		Yes	Yes	Yes	No	always	always
6	Yes	Yes	60		Yes	Yes	Yes	No	always	occasionally
7	Yes	No	30		Yes	Yes	Yes	No	always	always
8	No	Yes	15		Yes	Yes	Yes	Yes	always	always
9	No	Yes	40		Yes	Yes	Yes	Yes	occasionally	always
10	No	Yes	50		Yes	Yes	Yes	No	always	occasionally
11	Yes	Yes	40		Yes	Yes	Yes	No	always	always
12	Yes	Yes	10		Yes	No	No	Yes	occasionally	always
13	No	Yes	50		Yes	Yes	Yes	Yes	always	always
14	No	No	40		Yes	No	Yes	Yes	occasionally	rarely
15	Yes	Yes	30		Yes	Yes	Yes	No	always	occasionally
16	No	No	30		Yes	Yes	Yes	No	occasionally	occasionally
17	Yes	Yes	10		Yes	Yes	Yes	Yes	always	always
18	Yes	Yes	60		Yes	Yes	Yes	Yes	occasionally	always
19	Yes	Yes	40		Yes	Yes	Yes	No	occasionally	occasionally
20	No	No	80		Yes	Yes	Yes	No	always	occasionally
21	Yes	Yes	10		No	No	Yes	Yes	always	always
22	No	Yes	60		Yes	Yes	Yes	No	always	always
23	Yes	Yes	30		Yes	Yes	Yes	No	occasionally	occasionally
24	No	Yes	200		No	No	No	Yes	always	always
25	Yes	No	85		Yes	Yes	Yes	No	occasionally	always
26	Yes	Yes	200		Yes	Yes	Yes	No	always	always
27	Yes	No	20		Yes	Yes	Yes	No	always	occasionally
28	Yes	Yes	50		Yes	Yes	Yes	No	always	occasionally
29	Yes	No	10		Yes	No	Yes	No	always	always
30	Yes	Yes	60		Yes	Yes	Yes	Yes	occasionally	occasionally
31	No	Yes	20		Yes	Yes	No	Yes	always	always
32	Yes	Yes	50		Yes	Yes	Yes	No	always	occasionally
33	Yes	Yes	10		Yes	No	Yes	Yes	always	always
34	Yes	Yes	25		Yes	No	Yes	No	always	always

35	Yes	No	50	No	No	Yes	Yes	always	occasionally
36	No	Yes	100	Yes	Yes	Yes	Yes	always	always
37	Yes	Yes	60	Yes	Yes	Yes	No	always	always
38	Yes	No	200	Yes	Yes	Yes	No	occasionally	always
39	Yes	Yes	70	Yes	Yes	Yes	Yes	occasionally	rarely
40	Yes	No	10	Yes	Yes	Yes	No	always	occasionally
41	Yes	Yes	20	Yes	Yes	No	Yes	always	occasionally
42	Yes	No	45	No	Yes	Yes	Yes	occasionally	always
43	No	Yes	10	Yes	Yes	Yes	Yes	always	always
44	No	No	40	Yes	Yes	Yes	No	always	always
45	Yes	Yes	15	No	No	No	No	occasionally	always
46	No	Yes	88	Yes	Yes	Yes	Yes	always	occasionally
47	Yes	Yes	50	Yes	Yes	Yes	No	occasionally	occasionally
48	Yes	Yes	30	No	No	Yes	Yes	always	always
49	Yes	Yes	40	Yes	Yes	No	No	occasionally	occasionally
50	No	Yes	45	No	No	Yes	Yes	always	occasionally
51	No	Yes	50	Yes	Yes	Yes	No	occasionally	always
52	No	Yes	20	Yes	Yes	Yes	Yes	occasionally	always
53	Yes	Yes	20	No	Yes	No	No	always	occasionally
54	Yes	No	25	No	No	Yes	Yes	occasionally	occasionally
55	Yes	Yes	15	Yes	Yes	No	No	always	occasionally
56	No	Yes	20	No	Yes	Yes	No	always	always
57	Yes	Yes	40	Yes	Yes	Yes	No	always	always
58	No	Yes	60	No	Yes	Yes	Yes	occasionally	always
59	No	Yes	50	No	Yes	Yes	Yes	occasionally	always
60	Yes	No	40	Yes	Yes	Yes	No	always	always
61	Yes	No	10	Yes	Yes	Yes	No	occasionally	always
62	Yes	Yes	60	Yes	Yes	Yes	Yes	always	always
63	Yes	No	50	Yes	Yes	Yes	No	always	occasionally
64	Yes	Yes	30	No	Yes	Yes	Yes	always	occasionally
65	Yes	No	100	Yes	Yes	Yes	Yes	always	always
66	No	Yes	70	No	No	Yes	Yes	always	always
67	Yes	Yes	10	Yes	Yes	No	No	always	occasionally
68	Yes	No	50	Yes	Yes	Yes	No	occasionally	always
69	Yes	Yes	20	Yes	Yes	Yes	Yes	always	always

70	Yes	No	25	Yes	Yes	Yes	No	occasionally	always
71	No	Yes	150	Yes	Yes	Yes	No	occasionally	always
72	Yes	Yes	10	No	Yes	No	No	always	always
73	Yes	No	50	Yes	Yes	Yes	No	always	occasionally
74	Yes	Yes	10	Yes	Yes	Yes	No	always	always
75	No	Yes	20	No	Yes	Yes	Yes	occasionally	always
76	No	Yes	10	No	Yes	Yes	No	always	always
77	Yes	Yes	20	Yes	No	Yes	Yes	always	always
78	Yes	No	50	Yes	Yes	Yes	No	always	always
79	Yes	Yes	70	Yes	No	Yes	No	always	occasionally
80	Yes	Yes	50	Yes	Yes	Yes	No	always	always
81	No	Yes	30	Yes	Yes	Yes	Yes	always	always
82	Yes	Yes	10	No	Yes	Yes	No	occasionally	always
83	Yes	Yes	70	Yes	Yes	Yes	No	occasionally	always
84	Yes	No	90	Yes	Yes	Yes	No	always	always
85	Yes	No	10	No	Yes	Yes	No	always	always
86	Yes	No	30	No	No	Yes	Yes	always	always
87	No	Yes	30	Yes	Yes	Yes	Yes	occasionally	always
88	Yes	Yes	60	Yes	Yes	Yes	Yes	occasionally	always
89	No	Yes	20	Yes	Yes	Yes	Yes	always	always
90	No	Yes	50	Yes	Yes	Yes	No	always	always
91	Yes	Yes	45	Yes	Yes	Yes	Yes	occasionally	always
92	Yes	Yes	65	No	Yes	No	Yes	always	always
93	Yes	No	20	Yes	Yes	Yes	No	always	always
94	No	Yes	35	Yes	Yes	Yes	Yes	always	always
95	Yes	No	20	Yes	Yes	Yes	No	always	always
96	Yes	Yes	50	Yes	Yes	Yes	No	always	occasionally
97	No	Yes	70	No	No	No	No	always	always
98	No	Yes	36	No	No	No	Yes	always	occasionally
99	Yes	Yes	10	No	No	Yes	Yes	occasionally	occasionally
100	Yes	Yes	25	Yes	Yes	Yes	No	always	always
101	No	Yes	10	Yes	Yes	No	No	always	always
102	Yes	No	30	Yes	Yes	Yes	No	always	occasionally
103	Yes	Yes	50	Yes	Yes	Yes	Yes	always	always
104	No	Yes	20	Yes	Yes	No	No	occasionally	always

105	Yes	No	98	Yes	Yes	Yes	Yes	always	always
106	Yes	Yes	30	Yes	Yes	Yes	No	always	always
107	Yes	No	11	Yes	Yes	Yes	No	always	always
108	Yes	Yes	20	No	No	Yes	No	always	always
109	Yes	Yes	30	No	Yes	No	No	always	always
110	Yes	No	10	Yes	Yes	Yes	No	occasionally	always
111	No	Yes	25	Yes	Yes	Yes	No	always	occasionally
112	No	Yes	30	No	No	Yes	Yes	occasionally	occasionally
113	No	Yes	10	Yes	Yes	No	No	always	occasionally
114	No	Yes	20	Yes	Yes	Yes	No	always	always
115	Yes	Yes	16	Yes	Yes	Yes	No	occasionally	always
116	Yes	Yes	80	No	Yes	Yes	Yes	always	always
117	Yes	Yes	50	Yes	Yes	Yes	No	always	occasionally
118	No	Yes	50	Yes	Yes	Yes	No	always	always
119	No	Yes	100	Yes	Yes	Yes	No	occasionally	always
120	Yes	Yes	10	No	Yes	No	No	always	always
121	No	No	60	Yes	Yes	Yes	No	occasionally	always
122	No	No	12	Yes	Yes	No	No	always	always
123	No	Yes	100	No	No	Yes	Yes	always	always
124	No	No	10	Yes	Yes	No	Yes	occasionally	always
125	No	Yes	20	No	Yes	No	No	always	always
126	Yes	Yes	20	Yes	No	Yes	No	always	always
127	Yes	Yes	50	No	No	Yes	No	occasionally	occasionally
128	Yes	No	10	Yes	Yes	Yes	No	always	always
129	Yes	Yes	200	No	No	Yes	Yes	always	occasionally
130	Yes	No	45	Yes	Yes	Yes	No	always	always
131	Yes	Yes	15	Yes	Yes	Yes	No	occasionally	occasionally
132	Yes	Yes	25	No	No	No	Yes	occasionally	always
133	Yes	Yes	15	Yes	Yes	Yes	No	always	always
134	No	Yes	45	Yes	Yes	No	No	always	occasionally
135	Yes	No	10	Yes	Yes	Yes	No	always	always
136	No	Yes	45	No	No	Yes	No	occasionally	always
137	Yes	Yes	200	No	No	Yes	Yes	always	always
138	Yes	Yes	50	Yes	Yes	Yes	No	always	always
139	No	Yes	40	No	No	Yes	Yes	always	occasionally

140	Yes	No	40	No	Yes	Yes	Yes	always	occasionally
141	Yes	No	30	Yes	Yes	Yes	No	occasionally	always
142	Yes	No	20	Yes	Yes	Yes	No	occasionally	always
143	No	No	30	No	Yes	Yes	No	always	always
144	Yes	No	50	No	Yes	Yes	No	always	occasionally
145	Yes	Yes	10	No	No	No	Yes	always	always
146	No	Yes	15	Yes	Yes	No	Yes	always	occasionally
147	No	No	40	Yes	Yes	Yes	No	always	always
148	No	Yes	30	No	No	Yes	Yes	occasionally	always
149	Yes	Yes	20	No	No	Yes	No	occasionally	always
150	No	Yes	20	No	Yes	No	No	always	always
151	Yes	Yes	20	Yes	Yes	Yes	No	always	always
152	Yes	Yes	40	No	Yes	Yes	No	always	always
153	Yes	Yes	20	Yes	Yes	Yes	No	occasionally	always
154	Yes	Yes	15	No	No	Yes	Yes	always	always
155	No	Yes	200	No	Yes	No	No	always	always
156	Yes	Yes	20	Yes	Yes	Yes	Yes	occasionally	always
157	Yes	Yes	40	Yes	Yes	Yes	Yes	always	occasionally
158	No	Yes	50	No	No	No	No	occasionally	always
159	Yes	Yes	50	Yes	Yes	Yes	Yes	always	occasionally
160	No	Yes	60	Yes	Yes	Yes	Yes	always	always
161	No	Yes	180	Yes	Yes	Yes	Yes	always	always
162	Yes	No	60	Yes	Yes	Yes	No	always	always
163	No	Yes	35	Yes	No	Yes	Yes	always	occasionally
164	No	No	10	No	Yes	Yes	Yes	always	always
165	Yes	Yes	60	Yes	Yes	Yes	No	occasionally	always
166	No	Yes	15	Yes	Yes	Yes	No	occasionally	always
167	Yes	No	50	No	Yes	Yes	No	occasionally	occasionally
168	Yes	Yes	10	Yes	No	Yes	Yes	always	occasionally
169	Yes	No	10	No	Yes	Yes	No	always	always
170	Yes	No	10	Yes	No	Yes	No	occasionally	occasionally
171	Yes	Yes	20	Yes	Yes	Yes	Yes	always	always
172	Yes	No	30	Yes	Yes	Yes	No	always	always
173	No	Yes	10	No	No	Yes	Yes	always	occasionally
174	Yes	Yes	30	Yes	Yes	Yes	No	occasionally	always

175	No	No	30	No	Yes	Yes	Yes	always	occasionally
176	Yes	Yes	50	Yes	Yes	Yes	No	occasionally	always
177	No	Yes	40	Yes	Yes	Yes	Yes	occasionally	always
178	No	No	42	Yes	Yes	Yes	No	always	occasionally
179	Yes	No	50	No	No	Yes	Yes	always	always
180	No	Yes	12	Yes	Yes	No	No	occasionally	occasionally
181	Yes	No	30	No	No	Yes	No	occasionally	always
182	Yes	No	10	Yes	Yes	Yes	Yes	occasionally	occasionally
183	Yes	No	60	Yes	Yes	Yes	No	always	always
184	Yes	Yes	10	No	No	Yes	Yes	always	occasionally
185	No	Yes	30	Yes	Yes	No	No	always	always
186	Yes	Yes	70	No	Yes	Yes	Yes	always	always
187	No	Yes	150	No	Yes	No	Yes	occasionally	always
188	No	Yes	50	Yes	Yes	Yes	Yes	always	always
189	Yes	Yes	100	Yes	Yes	Yes	No	occasionally	occasionally
190	Yes	Yes	30	Yes	Yes	No	Yes	always	occasionally
191	Yes	No	10	Yes	Yes	Yes	No	occasionally	always
192	Yes	No	10	Yes	Yes	No	No	always	occasionally
193	No	Yes	10	Yes	Yes	Yes	No	occasionally	occasionally
194	Yes	No	100	Yes	Yes	Yes	No	always	always
195	Yes	Yes	10	No	No	Yes	Yes	always	always
196	Yes	Yes	20	Yes	Yes	Yes	No	always	occasionally
197	No	Yes	60	Yes	Yes	Yes	No	always	always
198	No	Yes	25	No	No	No	Yes	always	always
199	Yes	Yes	20	Yes	Yes	Yes	No	always	always
200	No	Yes	200	Yes	No	Yes	No	always	always
201	No	Yes	10	Yes	Yes	Yes	No	always	always
202	Yes	Yes	10	No	No	No	No	always	always
203	Yes	Yes	60	Yes	Yes	Yes	No	always	always
204	Yes	Yes	20	Yes	Yes	Yes	Yes	always	occasionally
205	No	Yes	60	Yes	Yes	Yes	Yes	occasionally	always
206	Yes	No	40	No	No	Yes	Yes	always	always
207	Yes	Yes	10	No	Yes	Yes	No	always	always
208	Yes	No	35	Yes	No	Yes	Yes	always	occasionally
209	No	Yes	40	Yes	Yes	Yes	No	always	occasionally

210	Yes	Yes	10	No	No	No	Yes	always	always
211	Yes	Yes	10	No	Yes	Yes	No	occasionally	always
212	Yes	Yes	200	Yes	No	Yes	Yes	always	always
213	No	No	80	Yes	Yes	Yes	No	always	occasionally
214	Yes	No	50	Yes	Yes	Yes	No	occasionally	rarely
215	Yes	Yes	60	Yes	Yes	Yes	No	always	occasionally
216	Yes	Yes	40	Yes	Yes	Yes	No	occasionally	always
217	Yes	Yes	70	Yes	Yes	Yes	No	occasionally	occasionally
218	Yes	No	20	Yes	No	Yes	No	occasionally	always
219	No	Yes	100	Yes	Yes	Yes	Yes	always	always
220	Yes	No	20	Yes	Yes	Yes	No	always	occasionally
221	Yes	No	20	No	No	Yes	Yes	occasionally	always
222	No	Yes	60	Yes	Yes	No	Yes	always	always
223	No	Yes	100	No	Yes	No	Yes	always	always
224	Yes	Yes	20	Yes	Yes	Yes	No	always	occasionally
225	Yes	Yes	80	Yes	Yes	Yes	Yes	always	always
226	No	No	15	No	No	No	No	always	always
227	No	Yes	50	Yes	Yes	No	Yes	occasionally	always
228	No	Yes	150	Yes	Yes	Yes	No	always	occasionally
229	No	Yes	190	No	No	Yes	Yes	occasionally	always
230	Yes	Yes	20	No	Yes	No	Yes	occasionally	always
231	No	Yes	30	No	No	Yes	No	occasionally	occasionally
232	Yes	No	150	Yes	Yes	Yes	No	always	occasionally
233	Yes	Yes	10	No	No	Yes	Yes	always	always
234	Yes	No	45	Yes	Yes	Yes	No	always	always
235	Yes	Yes	10	No	No	No	Yes	always	always
236	No	Yes	30	Yes	Yes	Yes	Yes	always	always
237	No	No	80	Yes	Yes	Yes	No	always	occasionally
238	Yes	Yes	60	Yes	Yes	Yes	No	occasionally	occasionally
239	Yes	Yes	17	Yes	Yes	Yes	Yes	occasionally	always
240	Yes	No	30	Yes	Yes	Yes	No	occasionally	always
241	Yes	Yes	20	No	Yes	Yes	Yes	always	always
242	No	No	30	Yes	Yes	Yes	No	occasionally	always
243	Yes	No	200	No	Yes	Yes	No	occasionally	always
244	Yes	Yes	150	Yes	Yes	Yes	No	always	occasionally

245	Yes	Yes	100	Yes	Yes	Yes	Yes	occasionally	occasionally
246	No	Yes	50	Yes	Yes	Yes	No	always	always
247	Yes	No	30	Yes	Yes	Yes	No	always	always
248	No	Yes	60	Yes	Yes	No	Yes	always	always
249	Yes	Yes	50	Yes	Yes	Yes	No	always	always
250	Yes	No	40	Yes	Yes	Yes	No	always	always
251	Yes	No	10	No	Yes	Yes	Yes	always	always
252	No	Yes	12	No	Yes	Yes	No	occasionally	always
253	Yes	Yes	70	Yes	Yes	Yes	No	occasionally	always
254	Yes	No	90	No	Yes	Yes	No	always	always
255	No	Yes	10	Yes	Yes	Yes	No	always	occasionally
256	Yes	No	100	Yes	Yes	Yes	No	always	always
257	Yes	Yes	10	No	No	Yes	Yes	always	always
258	Yes	No	80	Yes	No	Yes	No	occasionally	always
259	Yes	No	25	Yes	No	Yes	Yes	occasionally	always
260	Yes	No	100	Yes	Yes	Yes	No	always	occasionally
261	No	Yes	30	Yes	Yes	Yes	No	always	always
262	Yes	Yes	30	Yes	No	No	Yes	always	occasionally
263	No	No	30	Yes	Yes	Yes	No	occasionally	always
264	Yes	Yes	18	No	No	Yes	Yes	occasionally	always
265	Yes	Yes	50	Yes	Yes	Yes	Yes	always	occasionally
266	Yes	Yes	60	Yes	Yes	Yes	No	occasionally	always
267	No	No	50	Yes	Yes	Yes	No	occasionally	always
268	Yes	Yes	10	No	Yes	Yes	No	always	always
269	Yes	Yes	80	Yes	Yes	Yes	No	always	always
270	Yes	Yes	10	No	No	Yes	Yes	always	always
271	No	Yes	10	No	No	No	Yes	always	always
272	No	No	40	Yes	Yes	Yes	No	always	occasionally
273	Yes	Yes	100	Yes	Yes	Yes	Yes	always	occasionally
274	Yes	Yes	50	No	No	Yes	Yes	always	always
275	No	Yes	58	No	No	No	Yes	always	always
276	Yes	Yes	60	Yes	Yes	Yes	Yes	always	always
277	No	Yes	20	No	No	Yes	Yes	occasionally	occasionally
278	Yes	Yes	100	No	Yes	Yes	No	always	always
279	No	Yes	20	Yes	No	Yes	No	always	always

280	Yes	Yes	10	Yes	Yes	Yes	No	occasionally	occasionally
281	Yes	No	10	Yes	Yes	Yes	No	always	always
282	Yes	No	80	Yes	Yes	Yes	Yes	always	always
283	No	Yes	25	No	Yes	No	Yes	occasionally	always
284	Yes	Yes	50	Yes	Yes	Yes	Yes	occasionally	occasionally
285	Yes	No	30	Yes	Yes	Yes	No	always	occasionally
286	Yes	Yes	10	No	Yes	Yes	No	occasionally	always
287	Yes	Yes	10	No	No	Yes	Yes	always	always
288	No	Yes	15	No	No	No	Yes	always	always
289	No	Yes	13	No	No	No	No	always	always
290	Yes	No	010	Yes	Yes	Yes	No	always	occasionally
291	Yes	Yes	50	Yes	Yes	Yes	No	always	always
292	No	Yes	12	Yes	No	Yes	Yes	always	occasionally
293	Yes	Yes	30	No	Yes	Yes	Yes	always	always
294	Yes	Yes	200	No	No	Yes	Yes	occasionally	always
295	No	Yes	30	No	No	No	Yes	occasionally	always
296	Yes	Yes	70	Yes	Yes	Yes	Yes	always	always
297	Yes	Yes	40	Yes	Yes	Yes	No	always	always
298	Yes	Yes	50	Yes	Yes	Yes	No	occasionally	always
299	No	Yes	130	Yes	Yes	No	No	occasionally	always
300	No	No	60	Yes	Yes	Yes	No	always	always
301	Yes	Yes	60	Yes	Yes	No	Yes	always	always
302	Yes	Yes	80	No	No	Yes	No	occasionally	occasionally
303	Yes	Yes	30	No	No	No	Yes	always	occasionally
304	No	No	40	No	No	Yes	Yes	always	always
305	No	Yes	35	Yes	Yes	Yes	No	always	always
306	Yes	Yes	40	Yes	Yes	Yes	No	always	occasionally
307	Yes	Yes	10	Yes	Yes	Yes	No	always	occasionally
308	Yes	No	30	No	No	Yes	Yes	always	always
309	Yes	No	10	Yes	Yes	Yes	No	always	always
310	Yes	Yes	12	Yes	Yes	Yes	Yes	occasionally	always
311	Yes	Yes	10	No	Yes	Yes	No	always	always
312	Yes	Yes	10	No	Yes	No	Yes	always	always
313	Yes	Yes	30	Yes	Yes	Yes	No	occasionally	occasionally
314	No	Yes	10	Yes	Yes	Yes	No	always	occasionally

315	Yes	Yes	30	Yes	Yes	No	No	always	always
316	Yes	Yes	30	Yes	Yes	Yes	Yes	always	always
317	Yes	No	30	No	No	Yes	Yes	always	occasionally
318	Yes	Yes	20	Yes	Yes	Yes	Yes	occasionally	always
319	Yes	Yes	10	Yes	Yes	Yes	No	always	always
320	Yes	Yes	10	No	No	Yes	Yes	always	always
321	No	Yes	80	Yes	Yes	Yes	Yes	always	always
322	No	No	150	Yes	Yes	Yes	No	always	always
323	Yes	Yes	10	Yes	Yes	Yes	Yes	occasionally	always
324	No	Yes	50	Yes	Yes	Yes	Yes	occasionally	always
325	Yes	Yes	20	Yes	Yes	Yes	No	occasionally	occasionally
326	No	No	30	Yes	Yes	Yes	No	occasionally	always
327	No	Yes	10	No	Yes	Yes	No	always	always
328	No	Yes	60	Yes	Yes	Yes	No	occasionally	always
329	Yes	Yes	30	No	Yes	Yes	Yes	always	always
330	Yes	Yes	40	Yes	Yes	Yes	Yes	always	always
331	Yes	Yes	15	No	Yes	Yes	Yes	always	always
332	No	Yes	50	Yes	Yes	Yes	No	always	always
333	No	No	20	No	No	Yes	Yes	always	occasionally
334	Yes	No	30	Yes	Yes	No	No	always	always
335	Yes	Yes	40	Yes	Yes	Yes	No	always	always
336	No	Yes	50	No	No	No	No	always	occasionally
337	Yes	No	20	No	No	Yes	Yes	occasionally	always
338	Yes	Yes	50	Yes	Yes	Yes	No	always	always
339	No	Yes	30	No	Yes	Yes	Yes	always	always
340	No	Yes	30	Yes	Yes	Yes	No	occasionally	always
341	Yes	No	20	Yes	Yes	Yes	No	always	always
342	Yes	Yes	60	Yes	Yes	Yes	No	always	always
343	No	Yes	80	Yes	No	No	Yes	occasionally	occasionally
344	No	Yes	80	No	No	Yes	Yes	always	always
345	Yes	Yes	15	Yes	Yes	Yes	Yes	always	occasionally
346	Yes	Yes	15	Yes	Yes	Yes	No	always	always

Review patients	Assess the app	Assess the pati	Assess potentia	Establish mutua	Encourage the	Elicit patient's e	Discuss benefit	Discuss potenti	Inform patients
always	occasionally	occasionally	always	always	always	always	always	occasionally	occasionally
always	always	always	always	always	always	always	always	occasionally	occasionally
always	occasionally	always	occasionally	always	occasionally	always	occasionally	always	always
always	always	always	occasionally	always	occasionally	always	occasionally	occasionally	always
always	always	occasionally	always	always	occasionally	occasionally	occasionally	always	always
occasionally	always	always	always	always	occasionally	always	always	always	always
always	occasionally	always	always	occasionally	always	occasionally	occasionally	occasionally	occasionally
always	occasionally	always	always	occasionally	occasionally	always	always	always	occasionally
occasionally	occasionally	occasionally	always	always	occasionally	always	always	occasionally	always
always	always	occasionally	always	occasionally	occasionally	always	occasionally	occasionally	always
always	always	always	occasionally	always	occasionally	always	always	occasionally	always
always	always	always	always	always	occasionally	always	always	occasionally	occasionally
always	always	always	always	occasionally	always	always	always	always	always
always	always	always	occasionally	occasionally	occasionally	always	occasionally	always	occasionally
occasionally	always	occasionally	always	always	occasionally	always	always	occasionally	occasionally
always	always	occasionally	always	always	occasionally	always	occasionally	occasionally	occasionally
always	occasionally	occasionally	always	occasionally	always	always	occasionally	occasionally	always
occasionally	occasionally	always	always	always	always	always	occasionally	occasionally	occasionally
occasionally	always	always	occasionally	always	occasionally	always	always	occasionally	occasionally
occasionally	always	rarely	occasionally	always	occasionally	always	always	always	always
always	always	occasionally	occasionally	always	occasionally	occasionally	occasionally	occasionally	occasionally
always	occasionally	always	always	always	always	always	always	always	occasionally
always	always	always	occasionally	occasionally	occasionally	occasionally	always	always	always
occasionally	always	always	always	occasionally	always	always	occasionally	occasionally	always
always	occasionally	always	occasionally	always	always	occasionally	always	occasionally	always
always	always	occasionally	always	always	occasionally	occasionally	occasionally	always	always
always	always	always	occasionally	always	always	always	occasionally	always	always
occasionally	always	rarely	rarely	occasionally	always	occasionally	always	occasionally	always
always	occasionally	occasionally	occasionally	always	occasionally	always	occasionally	always	always
always	always	always	always	always	occasionally	occasionally	always	always	always
occasionally	always	always	occasionally	always	occasionally	always	always	always	occasionally
always	occasionally	occasionally	rarely	always	always	always	always	always	occasionally
always	always	occasionally	always	occasionally	always	always	always	occasionally	occasionally
occasionally	occasionally	always	always	always	always	occasionally	occasionally	always	occasionally

always	always	always	occasionally	occasionally	rarely	always	always	always	always
always	always	always	occasionally	always	occasionally	always	always	occasionally	always
always	occasionally	occasionally	always	always	occasionally	occasionally	always	always	always
always	occasionally	always	always	occasionally	always	occasionally	occasionally	always	always
occasionally	always	occasionally	occasionally	occasionally	always	always	always	always	occasionally
always	occasionally	always	always	occasionally	always	occasionally	occasionally	occasionally	always
always	always	occasionally	occasionally	always	always	occasionally	occasionally	occasionally	rarely
always	occasionally	occasionally	always	always	always	occasionally	always	occasionally	occasionally
always	always	occasionally	always	occasionally	always	occasionally	always	always	occasionally
always	occasionally	always	always	always	always	always	always	occasionally	occasionally
always	occasionally	always	always	always	occasionally	always	always	always	always
occasionally	occasionally	always	always	always	always	occasionally	occasionally	always	always
always	occasionally	always	always	occasionally	occasionally	always	always	occasionally	occasionally
always	always	always	always	occasionally	always	occasionally	always	always	occasionally
always	occasionally	occasionally	always	always	always	always	occasionally	always	occasionally
always	always	occasionally	occasionally	always	always	always	occasionally	always	always
always	always	always	always	occasionally	always	occasionally	occasionally	always	occasionally
occasionally	always	always	occasionally	always	always	always	always	always	occasionally
always	occasionally	occasionally	always	occasionally	always	occasionally	occasionally	occasionally	always
always	always	always	occasionally	rarely	occasionally	occasionally	occasionally	always	occasionally
occasionally	always	occasionally	always	occasionally	occasionally	always	occasionally	rarely	always
always	occasionally	always	always	occasionally	occasionally	rarely	occasionally	rarely	always
always	always	occasionally	always	always	always	always	occasionally	occasionally	always
always	always	occasionally	occasionally	occasionally	always	always	occasionally	occasionally	occasionally
always	always	always	always	always	always	occasionally	occasionally	always	always
always	occasionally	always	always	always	occasionally	always	always	always	occasionally
always	always	always	always	always	occasionally	occasionally	always	occasionally	always
always	always	always	always	always	always	always	always	occasionally	occasionally
always	occasionally	always	always	always	occasionally	occasionally	occasionally	occasionally	always
always	always	occasionally	always	always	always	always	always	always	always
always	occasionally	always	always	occasionally	always	always	always	always	occasionally
always	always	always	always	occasionally	always	always	occasionally	occasionally	always
occasionally	always	always	occasionally	occasionally	rarely	always	always	always	always
always	always	always	always	always	always	always	always	occasionally	occasionally
always	occasionally	occasionally	always	always	occasionally	always	always	always	always

always	occasionally	always	occasionally	occasionally	occasionally	always	always	always	occasionally
always	occasionally	always	always	occasionally	occasionally	always	always	occasionally	occasionally
always	always	always	always	always	occasionally	always	occasionally	occasionally	always
always	always	occasionally	always	occasionally	occasionally	occasionally	always	always	occasionally
occasionally	occasionally	occasionally	occasionally	always	always	occasionally	always	always	occasionally
always	occasionally	always	occasionally	always	always	occasionally	always	always	always
always	always	always	occasionally	always	always	occasionally	occasionally	occasionally	always
occasionally	always	occasionally	rarely	always	occasionally	always	always	always	occasionally
always	occasionally	always	always	occasionally	always	always	occasionally	always	occasionally
always	always	always	always	always	always	occasionally	always	occasionally	always
always	always	always	always	always	always	always	occasionally	always	always
always	occasionally	always	occasionally	rarely	rarely	always	always	always	always
always	occasionally	always	always	always	always	always	occasionally	always	always
always	always	occasionally	always	always	always	always	always	always	always
always	always	always	always	always	always	always	occasionally	occasionally	occasionally
always	occasionally	occasionally	always	always	always	always	occasionally	always	always
occasionally	always	always	always	always	always	always	occasionally	always	occasionally
occasionally	always	always	always	always	always	always	occasionally	always	occasionally
always	occasionally	always	always	always	always	occasionally	occasionally	occasionally	occasionally
always	always	always	always	always	always	occasionally	occasionally	always	always
always	always	occasionally	occasionally	always	always	occasionally	occasionally	occasionally	occasionally
always	occasionally	always	rarely	rarely	always	occasionally	always	always	always
always	always	always	always	always	occasionally	always	occasionally	always	always
always	always	always	always	always	always	always	always	always	always
always	occasionally	occasionally	always	always	always	always	always	always	occasionally
always	always	always	always	occasionally	occasionally	occasionally	always	always	always
occasionally	always	occasionally	always	occasionally	always	always	always	always	always
occasionally	always	occasionally	always	always	occasionally	always	always	always	always
always	occasionally	always	occasionally	always	always	occasionally	always	occasionally	occasionally
always	always	always	occasionally	always	occasionally	occasionally	always	always	occasionally
always	occasionally	occasionally	occasionally	always	occasionally	occasionally	occasionally	always	always
occasionally	always	occasionally	always	always	always	always	occasionally	always	always
always	always	occasionally	occasionally	always	rarely	always	occasionally	occasionally	rarely
always	occasionally	occasionally	always	occasionally	occasionally	always	occasionally	always	always
always	always	occasionally	occasionally	always	always	occasionally	always	always	always

always	occasionally	always	occasionally	rarely	occasionally	always	occasionally	occasionally	occasionally
always	occasionally	always	occasionally	always	occasionally	occasionally	always	always	always
always	always	always	always	always	always	occasionally	occasionally	always	always
always	always	always	occasionally	occasionally	always	occasionally	occasionally	always	always
always	always	always	always	occasionally	always	occasionally	occasionally	always	always
always	always	occasionally	occasionally	occasionally	always	occasionally	rarely	always	always
always	occasionally	occasionally	occasionally	occasionally	always	always	always	always	always
occasionally	occasionally	rarely	rarely	always	occasionally	rarely	rarely	occasionally	always
occasionally	always	always	always	always	always	occasionally	always	always	occasionally
always	always	occasionally	always	always	occasionally	occasionally	always	always	occasionally
always	occasionally	always	rarely	always	always	occasionally	always	occasionally	always
always	always	always	always	occasionally	occasionally	always	occasionally	always	always
occasionally	always	always	always	always	occasionally	always	occasionally	always	occasionally
occasionally	always	occasionally	rarely	always	always	occasionally	always	always	always
always	always	occasionally	always	always	occasionally	occasionally	occasionally	occasionally	always
always	occasionally	occasionally	always	always	occasionally	occasionally	occasionally	always	always
always	always	occasionally	occasionally	occasionally	rarely	always	occasionally	always	always
always	occasionally	always	always	always	occasionally	always	always	always	occasionally
always	always	occasionally	always	always	always	always	always	occasionally	always
always	always	always	occasionally	always	occasionally	always	always	occasionally	always
always	always	always	occasionally	always	always	always	occasionally	occasionally	always
always	occasionally	occasionally	always	always	occasionally	always	always	always	always
always	occasionally	always	occasionally	always	always	occasionally	occasionally	occasionally	always
occasionally	occasionally	occasionally	always	always	always	always	always	always	always
occasionally	occasionally	always	always	always	rarely	always	always	occasionally	always
always	always	occasionally	always	occasionally	always	always	always	occasionally	always
always	always	always	occasionally	always	always	occasionally	always	occasionally	occasionally
always	occasionally	always	occasionally	occasionally	always	always	occasionally	occasionally	always
always	occasionally	always	always	always	occasionally	occasionally	always	occasionally	always
always	occasionally	always	always	always	occasionally	always	always	occasionally	occasionally
always	occasionally	occasionally	occasionally	always	always	always	always	always	always
always	occasionally	always	occasionally	always	always	occasionally	always	occasionally	always
always	always	always	occasionally	occasionally	occasionally	always	always	always	always
always	occasionally	always	always	occasionally	occasionally	occasionally	occasionally	occasionally	always
always	always	always	always	occasionally	rarely	always	always	occasionally	occasionally

occasionally	always	occasionally	rarely	occasionally	always	always	always	occasionally	occasionally
always	occasionally	occasionally	occasionally	occasionally	always	always	always	always	always
always	occasionally	always	always	occasionally	rarely	rarely	always	always	always
occasionally	always	always	occasionally	always	always	occasionally	always	always	always
always	always	always	always	occasionally	always	occasionally	always	occasionally	always
always	always	occasionally	occasionally	always	occasionally	always	always	always	always
always	occasionally	occasionally	rarely	always	always	always	occasionally	occasionally	always
always	occasionally	always	always	occasionally	always	always	occasionally	always	occasionally
always	always	occasionally	always	occasionally	occasionally	occasionally	occasionally	rarely	rarely
always	always	occasionally	always	always	occasionally	occasionally	always	always	always
occasionally	always	occasionally	always	always	always	occasionally	occasionally	always	always
occasionally	always	always	always	always	always	always	occasionally	occasionally	occasionally
always	occasionally	always	always	occasionally	always	always	always	occasionally	always
always	occasionally	always	always	always	always	always	occasionally	occasionally	always
always	occasionally	always	always	always	occasionally	occasionally	always	occasionally	occasionally
always	always	occasionally	always	always	always	occasionally	always	always	occasionally
always	always	occasionally	always	occasionally	rarely	rarely	always	occasionally	rarely
always	always	always	always	always	occasionally	always	always	always	always
always	occasionally	occasionally	always	occasionally	always	occasionally	occasionally	always	occasionally
always	always	occasionally	always	always	occasionally	occasionally	always	occasionally	always
always	always	always	occasionally	rarely	always	occasionally	occasionally	occasionally	occasionally
always	always	always	occasionally	always	always	always	occasionally	occasionally	always
occasionally	always	occasionally	always	occasionally	always	always	always	occasionally	always
always	always	always	always	always	occasionally	always	occasionally	always	occasionally
occasionally	occasionally	occasionally	always	always	always	occasionally	occasionally	always	always
always	occasionally	occasionally	always	always	occasionally	always	occasionally	always	always
occasionally	always	always	always	occasionally	rarely	always	always	always	always
always	occasionally	occasionally	rarely	occasionally	rarely	occasionally	always	always	always
occasionally	always	always	occasionally	occasionally	always	occasionally	always	always	occasionally
always	always	occasionally	always	always	occasionally	always	occasionally	always	occasionally
always	always	occasionally	always	occasionally	always	occasionally	always	occasionally	occasionally
always	occasionally	always	occasionally	always	occasionally	occasionally	occasionally	always	occasionally
always	occasionally	always	always	occasionally	always	occasionally	occasionally	always	always
occasionally	occasionally	always	occasionally	rarely	always	always	occasionally	occasionally	always
occasionally	always	occasionally	always	occasionally	always	always	always	occasionally	occasionally

always	occasionally	occasionally	always	always	always	always	always	always	always
occasionally	always	always	occasionally	occasionally	rarely	always	always	always	always
always	occasionally	occasionally	always	always	always	occasionally	always	always	always
always	always	occasionally	occasionally	always	always	occasionally	always	always	always
always	always	occasionally	always	occasionally	always	always	occasionally	always	always
occasionally	always	always	occasionally	always	occasionally	always	always	always	occasionally
always	always	occasionally	always	occasionally	always	occasionally	always	always	always
occasionally	always	always	occasionally	occasionally	always	always	always	occasionally	always
always	always	always	always	occasionally	always	always	occasionally	occasionally	occasionally
always	always	occasionally	occasionally	occasionally	always	always	occasionally	always	always
always	always	occasionally	occasionally	always	always	always	occasionally	occasionally	always
always	always	always	occasionally	always	occasionally	always	always	occasionally	occasionally
always	always	always	always	occasionally	occasionally	occasionally	occasionally	occasionally	always
occasionally	always	occasionally	always	always	occasionally	always	always	occasionally	always
occasionally	always	occasionally	always	occasionally	always	occasionally	always	occasionally	occasionally
always	occasionally	occasionally	occasionally	rarely	occasionally	rarely	rarely	always	always
occasionally	always	always	always	occasionally	always	always	occasionally	occasionally	always
always	always	occasionally	always	occasionally	always	occasionally	always	occasionally	always
occasionally	occasionally	occasionally	always	occasionally	always	occasionally	occasionally	occasionally	occasionally
always	always	occasionally	always	occasionally	always	always	always	always	always
always	always	occasionally	always	always	occasionally	always	always	occasionally	occasionally
always	always	always	occasionally	occasionally	always	always	occasionally	always	occasionally
always	always	always	always	always	always	occasionally	occasionally	always	always
always	always	always	always	occasionally	occasionally	always	always	always	always
always	occasionally	always	always	always	occasionally	always	occasionally	occasionally	always
occasionally	always	always	always	always	occasionally	always	occasionally	always	always
always	occasionally	occasionally	always	always	always	occasionally	always	always	always
always	always	always	always	always	always	occasionally	occasionally	always	occasionally
always	always	always	always	always	always	always	always	always	always
always	always	always	occasionally	occasionally	occasionally	always	occasionally	always	occasionally
always	always	always	always	always	occasionally	always	always	always	occasionally
occasionally	always	always	occasionally	occasionally	always	occasionally	occasionally	occasionally	always
occasionally	occasionally	always	occasionally	always	always	always	occasionally	always	occasionally
occasionally	always	always	always	always	always	always	always	always	always
always	always	occasionally	rarely	occasionally	rarely	always	always	occasionally	always

always	always	occasionally	occasionally	always	always	always	always	occasionally	always
occasionally	always	always	occasionally	occasionally	always	always	always	occasionally	occasionally
occasionally	occasionally	always	always	occasionally	occasionally	always	always	always	occasionally
always	always	always	occasionally	always	always	always	occasionally	occasionally	rarely
occasionally	always	occasionally	always	always	always	always	always	occasionally	occasionally
always	always	always	always	always	always	always	always	always	always
always	occasionally	occasionally	always	always	always	always	always	occasionally	always
always	always	occasionally	occasionally	always	always	always	occasionally	always	occasionally
always	always	always	always	occasionally	always	occasionally	always	always	occasionally
always	always	always	always	always	occasionally	always	occasionally	always	occasionally
occasionally	always	occasionally	always	always	occasionally	occasionally	occasionally	always	always
always	occasionally	occasionally	always	occasionally	always	always	always	always	always
always	always	always	occasionally	always	occasionally	always	occasionally	always	occasionally
occasionally	rarely	occasionally	occasionally	always	always	always	always	always	occasionally
always	occasionally	always	occasionally	always	occasionally	always	always	always	occasionally
always	occasionally	always	occasionally	always	occasionally	always	occasionally	always	always
always	always	always	always	always	occasionally	always	always	occasionally	always
always	always	occasionally	occasionally	rarely	occasionally	always	occasionally	always	always
always	always	occasionally	rarely	always	occasionally	rarely	always	always	always
always	occasionally	occasionally	always	always	occasionally	always	always	occasionally	always
occasionally	occasionally	rarely	occasionally	always	occasionally	occasionally	always	occasionally	always
always	always	occasionally	occasionally	occasionally	occasionally	always	always	always	always
always	always	always	always	occasionally	always	occasionally	occasionally	occasionally	occasionally
always	always	always	always	always	always	always	always	occasionally	always
always	always	always	always	occasionally	always	always	occasionally	always	always
always	always	always	always	always	occasionally	occasionally	always	occasionally	always
always	occasionally	always	always	always	always	always	always	always	always
always	occasionally	always	always	occasionally	occasionally	occasionally	always	always	always
rarely	occasionally	occasionally	always	always	always	always	occasionally	occasionally	occasionally
always	always	occasionally	occasionally	occasionally	always	always	always	occasionally	occasionally
always	occasionally	always	occasionally	occasionally	always	occasionally	occasionally	always	always
occasionally	always	always	occasionally	occasionally	occasionally	always	occasionally	occasionally	always
occasionally	rarely	rarely	always	occasionally	always	always	always	occasionally	always
always	occasionally	occasionally	always	always	always	always	occasionally	always	always
occasionally	occasionally	always	always	always	occasionally	occasionally	always	occasionally	always

occasionally	rarely	always	occasionally	occasionally	occasionally	occasionally	occasionally	always	occasionally
occasionally	occasionally	always	occasionally	always	occasionally	always	always	occasionally	always
always	always	occasionally	always	occasionally	always	always	always	always	always
always	occasionally	always	always	always	occasionally	occasionally	always	occasionally	occasionally
occasionally	occasionally	always	occasionally	occasionally	always	always	occasionally	occasionally	always
always	occasionally	always	always	always	rarely	always	always	occasionally	occasionally
occasionally	always	always	always	always	occasionally	always	always	occasionally	occasionally
occasionally	always	always	occasionally	always	always	always	always	always	always
occasionally	occasionally	always	always	occasionally	rarely	always	always	always	occasionally
occasionally	occasionally	always	occasionally	occasionally	occasionally	always	occasionally	always	always
always	always	occasionally	occasionally	occasionally	occasionally	always	always	occasionally	always
always	always	occasionally	always	always	always	occasionally	occasionally	occasionally	always
occasionally	always	occasionally	rarely	occasionally	rarely	always	occasionally	always	occasionally
always	rarely	rarely	occasionally	always	occasionally	occasionally	occasionally	always	occasionally
always	occasionally	always	always	occasionally	rarely	always	always	always	always
always	always	occasionally	occasionally	always	occasionally	always	occasionally	occasionally	always
always	always	occasionally	rarely	always	always	occasionally	occasionally	occasionally	always
always	always	always	always	always	occasionally	always	always	occasionally	occasionally
occasionally	always	always	always	occasionally	occasionally	always	always	occasionally	always
always	occasionally	always	always	occasionally	always	always	occasionally	occasionally	always
occasionally	always	occasionally	always	occasionally	occasionally	always	occasionally	always	occasionally
always	always	always	occasionally	occasionally	always	occasionally	rarely	occasionally	always
rarely	occasionally	occasionally	always	always	always	always	always	always	always
always	always	always	occasionally	always	occasionally	always	always	occasionally	occasionally
occasionally	always	always	occasionally	always	occasionally	occasionally	occasionally	always	always
always	always	always	occasionally	always	occasionally	occasionally	occasionally	always	always
always	always	always	always	always	always	always	occasionally	always	occasionally
always	always	always	always	always	occasionally	always	always	always	always
always	occasionally	always	occasionally	rarely	occasionally	always	always	occasionally	always
always	occasionally	occasionally	occasionally	occasionally	always	always	occasionally	occasionally	always
always	always	occasionally	always	occasionally	always	occasionally	always	always	always
occasionally	always	occasionally	always	always	occasionally	always	occasionally	always	occasionally
always	occasionally	always	occasionally	rarely	always	always	always	occasionally	occasionally
occasionally	always	always	always	occasionally	occasionally	occasionally	always	always	always
occasionally	always	always	always	occasionally	always	occasionally	always	always	always

always	always	always	always	always	always	always	occasionally	always	occasionally
always	always	occasionally	occasionally	occasionally	rarely	always	always	always	always
occasionally	always	occasionally	always	always	occasionally	occasionally	always	always	always
always	always	always	occasionally	rarely	occasionally	occasionally	always	occasionally	always
always	occasionally	occasionally	always	occasionally	always	occasionally	always	always	always
occasionally	occasionally	occasionally	rarely	occasionally	always	always	always	occasionally	always
always	always	always	occasionally	always	occasionally	occasionally	always	occasionally	always
always	always	always	occasionally	always	occasionally	always	occasionally	occasionally	always
occasionally	always	occasionally	always	always	always	occasionally	occasionally	always	always
always	always	occasionally	occasionally	occasionally	always	occasionally	occasionally	always	occasionally
occasionally	always	occasionally	always	occasionally	always	occasionally	occasionally	always	occasionally
occasionally	always	always	always	always	always	always	always	always	occasionally
occasionally	always	always	occasionally	occasionally	always	always	occasionally	always	occasionally
rarely	always	always	always	always	always	always	always	occasionally	always
occasionally	occasionally	always	always	always	occasionally	occasionally	occasionally	always	occasionally
always	always	always	always	occasionally	occasionally	occasionally	rarely	occasionally	rarely
always	always	always	always	occasionally	occasionally	occasionally	always	occasionally	always
occasionally	always	always	always	always	always	occasionally	always	always	always
always	always	always	always	always	always	always	always	always	occasionally
always	always	always	always	always	always	always	always	always	always
always	always	occasionally	always	occasionally	always	always	always	always	occasionally
occasionally	always	occasionally	always	occasionally	always	occasionally	occasionally	always	occasionally
always	always	always	always	always	occasionally	occasionally	always	occasionally	occasionally
always	occasionally	always	occasionally	always	occasionally	occasionally	always	occasionally	always
occasionally	occasionally	always	always	occasionally	always	always	always	always	always
always	occasionally	occasionally	always	occasionally	rarely	occasionally	always	always	always
always	always	always	always	always	always	occasionally	always	occasionally	always
always	occasionally	occasionally	always	always	always	always	always	always	always
always	always	occasionally	always	occasionally	rarely	always	occasionally	always	always
always	occasionally	always	always	always	occasionally	occasionally	always	occasionally	occasionally
always	always	always	occasionally	occasionally	always	always	always	always	occasionally
always	always	always	always	always	occasionally	always	occasionally	always	always
always	always	always	always	occasionally	always	occasionally	always	occasionally	always
occasionally	rarely	rarely	occasionally	always	always	occasionally	occasionally	always	occasionally
occasionally	always	rarely	occasionally	occasionally	rarely	always	always	occasionally	always

occasionally	always	always	always	always	occasionally	always	always	always	occasionally
occasionally	always	always	occasionally	always	always	always	always	occasionally	occasionally
occasionally	always	always	occasionally	always	occasionally	always	occasionally	occasionally	always
always	always	occasionally	always	always	occasionally	always	always	occasionally	occasionally
occasionally	occasionally	always	always	occasionally	occasionally	always	occasionally	always	occasionally
always	always	occasionally	occasionally	rarely	always	occasionally	occasionally	occasionally	always
always	always	always	always	always	occasionally	occasionally	occasionally	always	always
always	always	occasionally	always	occasionally	occasionally	always	occasionally	occasionally	always
always	always	always	occasionally	occasionally	occasionally	always	always	always	always
always	occasionally	occasionally	always	always	always	always	occasionally	occasionally	always
always	always	always	always	always	always	always	always	always	occasionally
always	occasionally	always	always	occasionally	always	occasionally	occasionally	occasionally	always
always	always	occasionally	always	always	occasionally	always	always	always	always
occasionally	occasionally	always	always	occasionally	always	always	occasionally	always	occasionally
always	occasionally	occasionally	always	always	occasionally	always	always	occasionally	always
always	occasionally	always	always	occasionally	always	occasionally	always	always	always
always	occasionally	occasionally	always	always	always	occasionally	always	always	always
always	always	always	occasionally	occasionally	occasionally	occasionally	always	always	occasionally
always	always	always	always	always	occasionally	always	always	occasionally	always
always	always	occasionally	always	always	occasionally	always	always	always	occasionally
always	always	always	always	occasionally	always	always	always	always	occasionally
always	always	always	occasionally	always	always	always	occasionally	occasionally	rarely
always	occasionally	always	occasionally	occasionally	occasionally	occasionally	always	always	always
always	always	always	occasionally	always	occasionally	occasionally	always	always	occasionally
always	always	occasionally	always	always	occasionally	always	occasionally	always	always
occasionally	always	always	always	always	occasionally	occasionally	always	occasionally	occasionally
always	always	always	occasionally	always	always	always	always	always	always
occasionally	always	occasionally	occasionally	always	occasionally	always	always	always	occasionally
always	occasionally	always	always	occasionally	always	occasionally	occasionally	always	always
always	always	always	always	occasionally	occasionally	always	always	always	occasionally
occasionally	always	always	always	occasionally	always	always	always	always	always
always	occasionally	occasionally	occasionally	always	occasionally	always	occasionally	always	always

Provide alternat	Understand pat	Document the p	Inquire about th	Provide instruct	Provide instruct	Inform about th	Inform the patie	agement sc	Discuss the alte
always	occasionally	always	always	always	always	occasionally	always	33	agree
occasionally	occasionally	occasionally	always	always	occasionally	occasionally	always	31	agree
occasionally	always	always	occasionally	always	always	occasionally	always	33	agree
always	always	always	always	always	always	always	occasionally	35	strongly disagre
occasionally	always	always	always	always	occasionally	always	always	34	strongly agree
occasionally	always	always	always	occasionally	occasionally	always	occasionally	33	strongly agree
occasionally	always	occasionally	always	always	always	occasionally	always	31	agree
always	occasionally	always	always	occasionally	occasionally	occasionally	always	32	agree
always	occasionally	rarely	always	occasionally	occasionally	always	always	29	strongly agree
always	occasionally	always	occasionally	always	always	occasionally	always	31	strongly agree
occasionally	always	always	occasionally	occasionally	occasionally	occasionally	always	32	agree
always	rarely	always	occasionally	always	always	occasionally	occasionally	31	strongly agree
occasionally	always	occasionally	occasionally	occasionally	rarely	occasionally	occasionally	31	disagree
always	always	occasionally	rarely	occasionally	always	always	always	28	agree
occasionally	always	occasionally	always	always	always	always	occasionally	31	agree
always	occasionally	always	always	occasionally	always	occasionally	occasionally	29	agree
always	always	always	occasionally	occasionally	occasionally	rarely	occasionally	29	agree
occasionally	always	always	occasionally	always	always	always	always	32	disagree
always	occasionally	always	always	always	always	always	always	32	agree
occasionally	occasionally	always	always	always	always	occasionally	always	31	strongly agree
always	always	always	occasionally	always	always	always	occasionally	31	disagree
occasionally	always	always	always	always	always	always	occasionally	36	agree
always	always	occasionally	always	always	always	always	always	33	strongly agree
always	always	occasionally	always	occasionally	always	always	always	34	strongly agree
always	always	occasionally	always	always	occasionally	occasionally	always	32	strongly agree
occasionally	occasionally	always	always	always	occasionally	always	always	33	disagree
always	occasionally	always	always	occasionally	occasionally	occasionally	always	33	strongly agree
occasionally	rarely	always	always	occasionally	always	always	always	27	agree
always	occasionally	occasionally	rarely	always	always	always	occasionally	30	agree
occasionally	occasionally	always	occasionally	always	always	always	always	33	strongly agree
always	always	always	occasionally	always	always	always	occasionally	34	strongly agree
always	occasionally	always	occasionally	always	always	always	occasionally	31	agree
always	occasionally	always	always	occasionally	always	occasionally	always	33	agree
always	occasionally	occasionally	occasionally	always	always	occasionally	occasionally	30	agree

always	always	always	occasionally	always	always	always	always	34	agree
always	always	always	occasionally	occasionally	occasionally	always	always	34	disagree
always	always	always	always	always	always	always	always	36	agree
always	occasionally	occasionally	always	always	always	always	occasionally	32	strongly agree
always	always	always	always	always	always	always	occasionally	31	agree
always	always	always	always	always	always	always	always	34	agree
always	always	always	occasionally	always	always	always	occasionally	30	disagree
always	occasionally	always	always	always	occasionally	always	occasionally	31	agree
always	occasionally	occasionally	rarely	always	always	always	always	32	strongly agree
occasionally	always	occasionally	occasionally	occasionally	always	always	occasionally	32	disagree
always	occasionally	always	occasionally	always	always	occasionally	always	34	strongly agree
occasionally	always	always	always	always	occasionally	occasionally	always	32	strongly agree
occasionally	rarely	always	always	always	occasionally	always	occasionally	28	agree
occasionally	always	always	occasionally	occasionally	always	occasionally	occasionally	32	disagree
occasionally	always	always	always	occasionally	always	occasionally	occasionally	30	strongly agree
occasionally	always	always	occasionally	occasionally	always	always	always	33	agree
occasionally	always	always	always	always	occasionally	occasionally	occasionally	31	agree
occasionally	rarely	always	occasionally	always	always	always	always	32	strongly agree
occasionally	occasionally	always	always	occasionally	occasionally	always	always	29	agree
occasionally	always	always	always	always	always	always	always	30	agree
occasionally	occasionally	always	occasionally	occasionally	always	always	occasionally	27	strongly agree
always	always	always	occasionally	always	always	always	occasionally	30	strongly agree
occasionally	rarely	always	always	occasionally	occasionally	always	occasionally	31	agree
always	always	occasionally	occasionally	always	always	always	occasionally	30	agree
always	always	always	always	always	always	always	always	37	strongly agree
occasionally	occasionally	always	always	occasionally	occasionally	always	occasionally	32	agree
always	occasionally	always	always	occasionally	occasionally	always	always	33	agree
always	occasionally	occasionally	rarely	always	always	always	occasionally	33	strongly agree
always	occasionally	always	occasionally	always	always	occasionally	always	31	agree
occasionally	occasionally	always	occasionally	occasionally	always	always	occasionally	33	strongly agree
occasionally	occasionally	rarely	rarely	always	occasionally	always	occasionally	29	agree
always	occasionally	always	always	occasionally	occasionally	always	always	34	agree
always	always	occasionally	occasionally	occasionally	always	always	occasionally	30	strongly disagre
always	always	always	occasionally	always	occasionally	occasionally	rarely	32	strongly disagre
always	always	occasionally	occasionally	always	always	always	occasionally	34	strongly agree

always	always	always	occasionally	always	occasionally	always	always	32	agree
occasionally	rarely	occasionally	occasionally	occasionally	always	occasionally	always	27	agree
always	always	always	occasionally	always	occasionally	always	always	35	strongly agree
occasionally	occasionally	always	occasionally	occasionally	always	always	occasionally	29	agree
occasionally	occasionally	always	occasionally	always	always	always	always	31	agree
always	occasionally	always	occasionally	always	always	occasionally	occasionally	32	agree
always	always	occasionally	always	always	always	occasionally	always	34	strongly agree
always	occasionally	occasionally	always	occasionally	occasionally	occasionally	always	29	strongly agree
always	always	occasionally	occasionally	always	always	occasionally	always	33	agree
always	occasionally	occasionally	always	always	always	always	occasionally	34	strongly agree
always	always	always	always	occasionally	occasionally	always	occasionally	36	agree
occasionally	occasionally	always	occasionally	occasionally	occasionally	occasionally	always	28	strongly agree
always	rarely	always	occasionally	always	always	occasionally	always	33	strongly disagre
always	always	occasionally	always	occasionally	always	always	always	36	agree
always	always	always	occasionally	always	occasionally	always	always	35	agree
occasionally	always	always	always	always	always	always	occasionally	35	agree
always	always	always	always	always	always	always	always	37	strongly agree
occasionally	occasionally	always	occasionally	always	occasionally	occasionally	rarely	29	strongly agree
occasionally	rarely	always	occasionally	always	always	occasionally	occasionally	28	agree
occasionally	always	always	always	always	always	always	occasionally	36	strongly agree
occasionally	always	always	always	always	occasionally	always	always	32	agree
always	occasionally	always	always	occasionally	always	always	occasionally	30	disagree
always	always	always	always	always	always	occasionally	always	37	disagree
always	always	always	occasionally	always	occasionally	occasionally	always	37	agree
occasionally	rarely	always	occasionally	always	occasionally	always	occasionally	31	agree
always	always	always	occasionally	always	always	always	occasionally	35	agree
always	occasionally	occasionally	always	occasionally	always	occasionally	always	32	strongly disagre
always	always	always	occasionally	occasionally	occasionally	always	always	34	strongly agree
always	always	always	occasionally	occasionally	occasionally	always	always	31	agree
always	always	occasionally	always	always	occasionally	occasionally	always	31	agree
always	always	occasionally	occasionally	always	always	always	occasionally	31	strongly agree
occasionally	always	occasionally	occasionally	always	always	occasionally	occasionally	32	strongly agree
rarely	always	occasionally	always	occasionally	occasionally	always	occasionally	25	agree
always	occasionally	occasionally	occasionally	rarely	rarely	rarely	occasionally	25	agree
always	always	always	occasionally	always	always	always	occasionally	34	strongly agree

always	occasionally	occasionally	always	occasionally	always	always	occasionally	28	strongly agree
always	always	always	always	always	always	always	always	36	agree
always	occasionally	always	always	always	always	occasionally	always	36	strongly agree
always	always	always	always	always	always	always	occasionally	35	strongly disagre
always	occasionally	always	occasionally	always	always	occasionally	always	34	strongly agree
always	always	always	occasionally	always	always	always	occasionally	31	agree
occasionally	occasionally	always	occasionally	occasionally	always	always	occasionally	30	strongly agree
always	always	occasionally	occasionally	occasionally	always	occasionally	occasionally	21	agree
occasionally	rarely	always	occasionally	always	occasionally	always	occasionally	30	disagree
always	always	always	always	always	always	occasionally	occasionally	34	disagree
occasionally	rarely	always	occasionally	always	always	always	always	30	strongly agree
always	occasionally	always	occasionally	always	always	always	occasionally	34	agree
always	occasionally	always	always	occasionally	always	always	always	33	strongly agree
always	always	always	always	always	always	occasionally	occasionally	33	agree
occasionally	occasionally	always	always	always	always	always	occasionally	31	strongly agree
occasionally	rarely	always	occasionally	always	always	occasionally	occasionally	29	disagree
always	always	always	always	always	always	occasionally	always	32	strongly agree
always	occasionally	occasionally	occasionally	rarely	always	always	always	32	strongly agree
always	always	always	occasionally	occasionally	occasionally	always	occasionally	34	strongly disagre
always	always	always	occasionally	always	occasionally	occasionally	always	33	agree
occasionally	always	always	always	occasionally	always	always	always	35	agree
always	occasionally	occasionally	always	always	always	occasionally	occasionally	33	agree
always	occasionally	always	always	always	always	always	always	32	strongly agree
occasionally	always	occasionally	always	always	always	always	always	35	agree
always	always	always	always	occasionally	always	always	always	33	disagree
occasionally	always	occasionally	always	always	always	occasionally	occasionally	33	strongly agree
always	occasionally	always	always	occasionally	always	always	always	32	strongly agree
occasionally	rarely	always	always	occasionally	always	occasionally	occasionally	28	disagree
always	always	occasionally	always	always	always	always	always	35	disagree
always	occasionally	always	always	always	occasionally	always	always	33	strongly disagre
always	occasionally	occasionally	occasionally	always	always	occasionally	always	33	agree
occasionally	occasionally	always	always	always	occasionally	always	occasionally	31	strongly agree
occasionally	rarely	always	occasionally	occasionally	occasionally	always	always	31	agree
always	occasionally	always	always	occasionally	always	always	always	32	strongly agree
always	occasionally	always	always	always	always	always	always	33	agree

occasionally	always	occasionally	occasionally	always	occasionally	always	occasionally	27	strongly disagre
occasionally	always	always	occasionally	always	occasionally	always	always	32	agree
always	always	always	occasionally	always	always	occasionally	occasionally	30	agree
occasionally	always	occasionally	occasionally	always	always	always	occasionally	33	agree
always	occasionally	occasionally	always	always	occasionally	occasionally	always	32	strongly disagre
always	occasionally	occasionally	occasionally	always	occasionally	always	occasionally	32	agree
always	always	occasionally	occasionally	always	always	always	always	31	agree
always	always	always	always	occasionally	always	occasionally	occasionally	33	disagree
rarely	always	occasionally	occasionally	always	always	always	always	26	agree
occasionally	always	occasionally	occasionally	always	occasionally	always	always	32	agree
always	always	occasionally	always	always	occasionally	always	always	34	strongly disagre
always	occasionally	always	occasionally	occasionally	always	always	always	33	strongly agree
always	occasionally	always	occasionally	always	always	occasionally	always	34	strongly agree
occasionally	occasionally	always	always	always	always	occasionally	always	33	agree
always	always	always	occasionally	always	always	occasionally	always	33	disagree
always	always	always	occasionally	always	always	always	occasionally	35	agree
always	occasionally	occasionally	occasionally	always	occasionally	always	occasionally	25	agree
occasionally	occasionally	always	always	always	occasionally	occasionally	always	34	agree
occasionally	rarely	occasionally	occasionally	rarely	always	occasionally	occasionally	24	disagree
always	occasionally	occasionally	always	always	occasionally	occasionally	occasionally	30	agree
always	occasionally	always	occasionally	always	always	always	occasionally	30	strongly agree
occasionally	rarely	always	always	always	always	occasionally	occasionally	32	disagree
occasionally	rarely	always	occasionally	always	occasionally	occasionally	always	30	agree
always	always	always	always	always	occasionally	occasionally	occasionally	33	strongly agree
always	always	always	always	always	always	always	always	35	agree
always	occasionally	occasionally	rarely	always	always	always	occasionally	30	strongly agree
always	occasionally	always	always	occasionally	occasionally	occasionally	occasionally	30	strongly agree
always	always	always	always	always	always	always	always	30	agree
always	always	occasionally	always	occasionally	always	always	always	32	strongly agree
always	occasionally	always	occasionally	occasionally	always	occasionally	occasionally	31	strongly disagre
always	always	always	occasionally	always	always	always	always	32	strongly agree
always	occasionally	occasionally	always	always	always	occasionally	always	31	strongly agree
occasionally	always	always	occasionally	occasionally	always	occasionally	always	32	disagree
occasionally	occasionally	rarely	always	occasionally	occasionally	always	always	26	strongly agree
occasionally	occasionally	always	always	always	occasionally	always	occasionally	30	disagree

occasionally	always	always	always	always	always	always	occasionally	35	agree
always	always	always	always	always	occasionally	always	always	33	agree
always	occasionally	occasionally	always	occasionally	always	occasionally	always	32	disagree
always	always	always	occasionally	occasionally	always	always	always	34	agree
always	always	always	always	always	occasionally	occasionally	occasionally	34	agree
always	occasionally	always	always	always	occasionally	occasionally	always	31	agree
occasionally	occasionally	always	occasionally	occasionally	always	always	always	32	disagree
always	always	occasionally	always	occasionally	occasionally	always	always	31	agree
always	occasionally	occasionally	always	always	occasionally	rarely	occasionally	30	agree
occasionally	occasionally	always	always	always	always	always	occasionally	32	strongly disagre
occasionally	always	always	always	always	occasionally	occasionally	always	33	agree
always	occasionally	always	occasionally	always	always	always	always	34	agree
always	always	occasionally	occasionally	always	occasionally	always	always	31	agree
always	occasionally	always	always	occasionally	occasionally	occasionally	always	32	disagree
always	occasionally	occasionally	rarely	always	occasionally	occasionally	always	26	strongly agree
always	occasionally	occasionally	always	occasionally	occasionally	occasionally	always	24	strongly agree
always	occasionally	occasionally	always	always	always	always	always	33	strongly agree
occasionally	always	always	occasionally	always	occasionally	always	always	32	strongly agree
always	always	always	always	always	always	always	always	30	agree
always	occasionally	always	occasionally	always	always	always	occasionally	35	agree
always	occasionally	rarely	rarely	always	always	occasionally	occasionally	29	agree
occasionally	always	occasionally	rarely	occasionally	always	always	occasionally	29	disagree
occasionally	always	always	always	occasionally	always	always	occasionally	35	strongly agree
always	occasionally	always	always	always	always	always	always	37	disagree
occasionally	always	occasionally	always	always	always	always	always	34	strongly agree
occasionally	occasionally	always	always	always	occasionally	occasionally	occasionally	32	agree
always	always	always	occasionally	always	always	always	occasionally	35	agree
always	occasionally	occasionally	always	occasionally	occasionally	always	occasionally	32	agree
always	always	always	always	always	occasionally	always	occasionally	38	agree
always	occasionally	always	always	always	always	always	occasionally	32	disagree
always	occasionally	occasionally	always	always	occasionally	occasionally	rarely	31	strongly agree
always	occasionally	always	occasionally	always	always	occasionally	always	31	strongly agree
always	always	occasionally	always	always	always	occasionally	always	33	strongly agree
always	always	always	always	always	occasionally	always	always	37	agree
always	occasionally	always	always	always	occasionally	always	occasionally	29	agree

always	occasionally	always	always	always	occasionally	occasionally	occasionally	33	agree
always	occasionally	always	occasionally	always	always	occasionally	always	31	agree
occasionally	rarely	occasionally	rarely	always	occasionally	always	occasionally	27	agree
occasionally	always	always	occasionally	occasionally	always	occasionally	always	30	disagree
always	always	occasionally	occasionally	always	always	always	occasionally	30	agree
always	occasionally	always	always	always	occasionally	occasionally	always	36	agree
always	occasionally	occasionally	always	occasionally	occasionally	always	always	32	agree
always	occasionally	occasionally	occasionally	occasionally	occasionally	occasionally	always	28	strongly agree
occasionally	rarely	occasionally	always	occasionally	always	always	always	31	agree
occasionally	rarely	rarely	rarely	always	occasionally	always	occasionally	28	strongly agree
occasionally	occasionally	always	occasionally	always	occasionally	occasionally	always	29	strongly agree
occasionally	occasionally	occasionally	occasionally	always	occasionally	occasionally	occasionally	29	strongly disagre
occasionally	occasionally	always	always	always	always	always	occasionally	33	strongly agree
always	always	always	always	always	always	always	always	34	disagree
occasionally	occasionally	occasionally	occasionally	occasionally	always	always	occasionally	29	disagree
occasionally	occasionally	always	occasionally	always	always	occasionally	always	32	agree
always	always	occasionally	rarely	always	always	always	occasionally	34	agree
always	always	always	always	always	always	always	always	33	strongly agree
occasionally	rarely	always	occasionally	always	occasionally	always	occasionally	27	agree
always	always	occasionally	always	always	occasionally	always	always	33	disagree
always	occasionally	occasionally	always	occasionally	occasionally	always	rarely	25	strongly agree
always	occasionally	always	always	always	occasionally	always	always	32	strongly agree
occasionally	rarely	always	always	always	always	occasionally	always	30	strongly agree
always	always	occasionally	occasionally	occasionally	occasionally	rarely	occasionally	32	strongly agree
always	occasionally	occasionally	always	occasionally	occasionally	always	occasionally	33	strongly agree
always	occasionally	occasionally	always	always	occasionally	occasionally	occasionally	32	agree
always	always	occasionally	always	occasionally	always	occasionally	always	36	agree
always	occasionally	always	always	always	occasionally	always	always	33	agree
always	occasionally	always	always	occasionally	always	occasionally	always	28	agree
occasionally	rarely	occasionally	rarely	always	always	occasionally	always	27	strongly agree
occasionally	always	always	always	always	occasionally	always	occasionally	31	disagree
always	always	always	occasionally	always	occasionally	always	always	32	agree
always	occasionally	always	occasionally	always	always	occasionally	occasionally	28	strongly agree
always	occasionally	occasionally	always	always	always	always	always	34	strongly agree
always	always	occasionally	always	always	always	always	occasionally	32	agree

occasionally	always	always	occasionally	always	always	occasionally	always	26	agree
always	always	always	always	always	always	occasionally	occasionally	33	agree
occasionally	always	occasionally	occasionally	always	occasionally	always	always	34	agree
always	occasionally	always	occasionally	occasionally	always	occasionally	occasionally	30	strongly agree
always	always	always	occasionally	always	occasionally	always	always	32	agree
occasionally	always	occasionally	rarely	always	always	occasionally	always	30	strongly agree
always	occasionally	occasionally	occasionally	always	always	occasionally	occasionally	31	agree
occasionally	occasionally	always	always	occasionally	always	occasionally	always	33	strongly agree
always	always	always	occasionally	occasionally	always	always	always	31	strongly agree
occasionally	rarely	always	always	always	occasionally	occasionally	occasionally	28	agree
occasionally	always	always	always	always	always	occasionally	occasionally	31	strongly agree
always	always	occasionally	always	always	occasionally	always	occasionally	33	agree
occasionally	occasionally	always	occasionally	always	always	always	always	28	agree
always	always	always	always	always	occasionally	always	always	29	strongly agree
always	occasionally	always	occasionally	always	always	always	always	33	strongly agree
occasionally	occasionally	always	occasionally	always	always	occasionally	always	30	disagree
occasionally	always	always	occasionally	always	always	always	occasionally	31	disagree
always	occasionally	occasionally	always	always	occasionally	always	always	33	strongly agree
occasionally	always	always	always	always	always	always	always	34	disagree
occasionally	always	always	occasionally	always	always	always	occasionally	32	strongly agree
occasionally	always	always	always	always	always	always	always	32	agree
occasionally	occasionally	occasionally	occasionally	always	always	occasionally	always	28	agree
always	occasionally	always	always	always	always	always	always	34	strongly agree
always	occasionally	always	always	always	always	always	occasionally	34	agree
always	occasionally	always	occasionally	always	occasionally	always	always	32	agree
always	always	always	occasionally	occasionally	always	always	always	34	agree
always	occasionally	always	occasionally	always	always	always	always	36	strongly agree
occasionally	always	occasionally	always	always	always	occasionally	occasionally	34	agree
always	occasionally	always	occasionally	always	occasionally	always	always	30	disagree
always	occasionally	occasionally	always	occasionally	always	occasionally	occasionally	29	disagree
always	always	always	always	always	always	always	always	37	agree
always	occasionally	occasionally	always	occasionally	occasionally	occasionally	always	30	strongly agree
occasionally	occasionally	occasionally	rarely	always	always	always	always	27	strongly agree
occasionally	rarely	always	occasionally	occasionally	always	always	always	31	disagree
occasionally	always	always	always	occasionally	always	always	occasionally	34	strongly agree

always	occasionally	always	always	always	always	always	always	35	agree
always	occasionally	always	always	always	occasionally	occasionally	always	32	agree
occasionally	always	always	always	always	always	occasionally	occasionally	33	strongly agree
always	occasionally	occasionally	always	always	always	occasionally	occasionally	29	agree
occasionally	always	occasionally	occasionally	occasionally	occasionally	rarely	always	27	agree
always	always	always	occasionally	occasionally	occasionally	always	always	29	strongly agree
always	always	always	always	always	occasionally	always	always	34	strongly agree
always	occasionally	occasionally	always	always	always	occasionally	occasionally	32	agree
always	always	occasionally	always	always	always	always	occasionally	34	agree
occasionally	always	always	occasionally	always	always	occasionally	always	31	disagree
always	always	occasionally	occasionally	occasionally	occasionally	always	occasionally	28	strongly agree
always	occasionally	always	always	always	always	always	occasionally	36	strongly agree
always	occasionally	always	occasionally	occasionally	always	always	always	31	agree
always	occasionally	always	occasionally	always	always	always	always	35	agree
occasionally	occasionally	always	always	occasionally	always	always	always	30	strongly agree
always	occasionally	always	occasionally	occasionally	always	always	occasionally	27	agree
always	always	occasionally	always	always	occasionally	occasionally	always	33	strongly agree
always	occasionally	always	always	occasionally	always	occasionally	occasionally	34	strongly agree
occasionally	always	always	always	occasionally	always	occasionally	always	35	strongly agree
occasionally	always	occasionally	always	occasionally	always	always	always	36	disagree
always	occasionally	always	occasionally	occasionally	occasionally	rarely	rarely	29	strongly agree
always	occasionally	occasionally	always	occasionally	always	always	always	31	agree
always	occasionally	occasionally	always	always	always	always	always	32	strongly agree
occasionally	occasionally	occasionally	rarely	always	occasionally	occasionally	always	27	agree
always	occasionally	always	occasionally	occasionally	occasionally	rarely	always	31	agree
occasionally	occasionally	always	occasionally	always	occasionally	always	always	30	strongly agree
occasionally	occasionally	always	always	occasionally	occasionally	always	always	33	agree
always	always	always	occasionally	always	always	always	always	36	strongly agree
always	always	occasionally	occasionally	always	occasionally	occasionally	always	31	strongly agree
always	rarely	always	always	always	occasionally	always	always	32	disagree
occasionally	always	always	always	occasionally	occasionally	rarely	always	31	agree
occasionally	always	always	always	always	always	always	occasionally	36	agree
occasionally	occasionally	occasionally	occasionally	always	always	always	always	33	strongly agree
always	occasionally	always	occasionally	always	always	always	occasionally	26	strongly disagre
always	always	always	always	always	occasionally	occasionally	always	29	agree

always	occasionally	always	always	always	always	always	always	36	agree
always	always	occasionally	occasionally	always	occasionally	occasionally	always	32	agree
always	occasionally	always	always	always	occasionally	occasionally	always	31	agree
always	always	occasionally	occasionally	occasionally	always	always	occasionally	31	agree
occasionally	rarely	always	always	occasionally	occasionally	always	always	29	agree
always	always	always	always	always	always	always	always	33	strongly agree
always	always	occasionally	always	always	always	occasionally	always	35	agree
always	always	occasionally	rarely	always	always	occasionally	always	31	agree
always	occasionally	occasionally	occasionally	always	always	always	always	33	disagree
occasionally	always	occasionally	occasionally	always	always	always	always	32	strongly agree
occasionally	rarely	always	occasionally	occasionally	always	occasionally	always	31	strongly agree
occasionally	always	always	occasionally	occasionally	occasionally	occasionally	rarely	27	strongly agree
always	always	always	occasionally	always	always	occasionally	always	36	disagree
always	occasionally	always	always	always	always	occasionally	always	32	strongly agree
always	occasionally	always	always	always	always	always	always	35	agree
always	occasionally	occasionally	rarely	always	occasionally	always	always	32	agree
always	occasionally	always	always	always	always	occasionally	occasionally	34	agree
always	always	occasionally	occasionally	always	always	always	always	33	strongly agree
occasionally	always	always	occasionally	occasionally	always	occasionally	always	33	agree
occasionally	occasionally	occasionally	always	always	always	occasionally	always	33	strongly agree
occasionally	occasionally	occasionally	always	always	always	always	always	35	agree
always	always	occasionally	always	always	always	always	always	33	agree
occasionally	occasionally	always	always	always	always	occasionally	occasionally	30	agree
always	always	occasionally	always	always	occasionally	always	always	34	strongly agree
always	always	always	always	always	always	occasionally	always	36	agree
occasionally	occasionally	always	always	occasionally	always	always	always	31	strongly agree
always	occasionally	occasionally	rarely	always	always	always	always	35	strongly agree
always	always	occasionally	always	always	always	always	always	34	strongly agree
occasionally	occasionally	always	always	occasionally	always	occasionally	always	30	agree
always	always	occasionally	always	always	always	occasionally	occasionally	34	disagree
occasionally	always	occasionally	always	always	always	always	always	35	strongly agree
always	always	occasionally	always	occasionally	always	always	occasionally	32	agree

Understand the	Balance the trea	Identify drug-dru	Explain potentia	Handle the pote	Determine that	Unable to make new decisions due to clinical inertia
disagree	strongly agree	strongly disagre	disagree	agree	disagree	disagree
agree	agree	strongly disagre	strongly agree	agree	agree	disagree
strongly agree	disagree	agree	agree	strongly agree	agree	agree
disagree	disagree	strongly agree	strongly agree	agree	disagree	strongly disagree
disagree	disagree	strongly agree	disagree	disagree	disagree	disagree
agree	agree	agree	agree	agree	disagree	disagree
disagree	strongly agree	disagree	disagree	strongly agree	strongly disagre	disagree
agree	agree	disagree	strongly agree	strongly agree	strongly agree	strongly disagree
strongly agree	agree	strongly agree	disagree	disagree	agree	disagree
agree	strongly disagre	agree	disagree	disagree	strongly agree	disagree
agree	agree	disagree	disagree	disagree	agree	agree
strongly disagre	disagree	strongly agree	strongly disagre	disagree	agree	disagree
strongly agree	agree	strongly agree	strongly agree	agree	disagree	agree
disagree	disagree	strongly agree	disagree	disagree	agree	disagree
strongly agree	strongly agree	strongly agree	agree	strongly agree	strongly disagre	disagree
agree	strongly agree	disagree	agree	strongly agree	disagree	agree
strongly agree	agree	disagree	strongly agree	disagree	disagree	disagree
disagree	disagree	agree	agree	disagree	disagree	strongly disagree
agree	strongly agree	agree	strongly agree	strongly agree	agree	agree
agree	disagree	agree	disagree	agree	strongly agree	disagree
agree	agree	disagree	agree	strongly agree	disagree	disagree
agree	agree	disagree	agree	agree	agree	strongly disagree
agree	strongly agree	disagree	disagree	strongly agree	strongly agree	disagree
agree	agree	disagree	agree	agree	disagree	strongly disagree
disagree	disagree	agree	strongly agree	agree	disagree	strongly agree
disagree	disagree	disagree	strongly agree	disagree	agree	disagree
strongly agree	disagree	disagree	disagree	strongly agree	strongly agree	disagree
strongly agree	strongly agree	agree	agree	disagree	disagree	agree
agree	disagree	agree	agree	disagree	disagree	disagree
strongly disagre	disagree	disagree	strongly agree	disagree	agree	agree
agree	disagree	disagree	strongly agree	disagree	disagree	disagree
strongly agree	strongly agree	agree	agree	disagree	disagree	disagree
strongly agree	disagree	disagree	strongly agree	agree	disagree	strongly disagree
agree	strongly agree	agree	agree	agree	strongly agree	strongly agree

strongly agree	agree	agree	disagree	strongly agree	strongly agree	disagree
disagree	agree	disagree	agree	disagree	agree	disagree
agree	disagree	disagree	strongly agree	disagree	agree	disagree
agree	strongly agree	agree	agree	agree	disagree	strongly disagree
strongly agree	disagree	disagree	strongly agree	strongly agree	agree	disagree
strongly agree	disagree	disagree	strongly disagre	strongly agree	disagree	disagree
agree	strongly disagre	strongly disagre	disagree	agree	agree	strongly disagree
strongly agree	disagree	disagree	agree	strongly agree	strongly agree	strongly agree
strongly agree	agree	strongly disagre	agree	agree	strongly agree	disagree
strongly agree	agree	agree	agree	disagree	disagree	agree
agree	agree	strongly agree	strongly agree	agree	agree	disagree
agree	disagree	disagree	disagree	disagree	agree	disagree
strongly agree	disagree	agree	agree	strongly agree	disagree	disagree
strongly agree	disagree	agree	strongly agree	strongly agree	disagree	strongly disagree
strongly disagre	agree	agree	strongly disagre	disagree	agree	disagree
agree	strongly agree	agree	disagree	agree	disagree	strongly disagree
strongly agree	disagree	disagree	disagree	disagree	disagree	disagree
strongly agree	strongly agree	strongly disagre	disagree	agree	strongly agree	agree
agree	disagree	disagree	agree	agree	agree	agree
strongly agree	disagree	disagree	disagree	strongly agree	agree	agree
strongly agree	agree	strongly agree	disagree	strongly agree	strongly agree	agree
disagree	disagree	strongly agree	agree	strongly agree	agree	disagree
agree	agree	disagree	strongly agree	agree	disagree	disagree
strongly agree	agree	agree	disagree	strongly agree	disagree	strongly agree
strongly agree	strongly agree	disagree	agree	agree	disagree	disagree
strongly agree	agree	disagree	disagree	disagree	strongly disagre	disagree
agree	strongly agree	agree	disagree	agree	agree	disagree
strongly disagre	strongly agree	agree	disagree	strongly agree	agree	disagree
disagree	agree	disagree	agree	strongly agree	disagree	agree
strongly disagre	agree	agree	strongly agree	disagree	agree	agree
agree	disagree	agree	agree	strongly agree	disagree	disagree
agree	disagree	agree	strongly agree	agree	strongly agree	disagree
strongly agree	agree	agree	agree	disagree	agree	strongly disagree
agree	strongly disagre	disagree	disagree	strongly agree	strongly disagre	strongly disagree
agree	disagree	disagree	strongly agree	strongly agree	disagree	strongly disagree

strongly agree	agree	agree	disagree	strongly agree	agree	strongly disagree
strongly agree	disagree	strongly agree	disagree	agree	disagree	strongly disagree
strongly agree	disagree	disagree	strongly agree	agree	strongly agree	disagree
agree	strongly agree	disagree	agree	agree	disagree	agree
agree	agree	strongly agree	disagree	disagree	strongly disagre	agree
agree	agree	strongly agree	disagree	disagree	disagree	strongly agree
agree	agree	agree	agree	strongly agree	disagree	agree
agree	strongly agree	disagree	disagree	agree	agree	strongly disagree
strongly agree	strongly agree	strongly agree	disagree	strongly agree	disagree	disagree
agree	agree	agree	agree	strongly agree	agree	agree
strongly agree	disagree	strongly agree	agree	agree	strongly agree	disagree
agree	disagree	strongly agree	disagree	agree	agree	agree
strongly agree	strongly agree	strongly agree	agree	strongly agree	disagree	strongly agree
disagree	agree	agree	agree	disagree	strongly agree	disagree
disagree	disagree	agree	agree	agree	strongly agree	agree
agree	disagree	agree	strongly agree	agree	agree	strongly disagree
disagree	disagree	agree	agree	agree	disagree	agree
agree	strongly agree	agree	disagree	agree	disagree	strongly disagree
strongly agree	strongly agree	disagree	agree	agree	disagree	disagree
strongly agree	agree	disagree	agree	strongly agree	strongly disagre	disagree
strongly agree	disagree	strongly agree	agree	disagree	strongly agree	strongly agree
disagree	agree	agree	agree	disagree	strongly agree	strongly disagree
agree	agree	disagree	agree	disagree	strongly agree	disagree
strongly agree	strongly agree	disagree	agree	agree	agree	disagree
strongly agree	agree	disagree	disagree	strongly agree	strongly disagre	strongly disagree
strongly agree	disagree	strongly agree	disagree	disagree	disagree	strongly disagree
agree	disagree	agree	strongly disagre	disagree	agree	disagree
agree	agree	strongly disagre	agree	agree	strongly agree	disagree
strongly agree	agree	disagree	agree	disagree	disagree	strongly disagree
agree	agree	agree	disagree	strongly agree	disagree	disagree
strongly disagre	agree	strongly agree	agree	disagree	disagree	agree
strongly agree	agree	agree	agree	strongly agree	strongly agree	disagree
agree	agree	strongly disagre	agree	agree	disagree	agree
agree	agree	disagree	agree	disagree	disagree	disagree
agree	agree	disagree	agree	strongly agree	agree	disagree

agree	agree	disagree	agree	disagree	agree	strongly disagree
agree	agree	agree	agree	disagree	disagree	strongly disagree
disagree	disagree	agree	disagree	disagree	disagree	disagree
agree	agree	strongly agree	agree	strongly agree	strongly agree	disagree
agree	agree	disagree	agree	agree	agree	strongly agree
agree	agree	agree	strongly agree	disagree	disagree	strongly disagree
disagree	strongly agree	strongly agree	strongly agree	agree	strongly agree	agree
agree	disagree	disagree	disagree	agree	agree	strongly disagree
agree	strongly agree	strongly disagre	strongly disagre	agree	disagree	disagree
strongly disagre	disagree	strongly agree	disagree	strongly agree	agree	agree
agree	agree	disagree	strongly agree	strongly agree	agree	strongly disagree
strongly agree	agree	strongly agree	agree	disagree	agree	strongly disagree
disagree	disagree	strongly disagre	strongly agree	disagree	strongly agree	agree
agree	agree	disagree	strongly agree	agree	disagree	disagree
agree	agree	disagree	agree	agree	strongly agree	strongly disagree
strongly agree	disagree	agree	strongly agree	strongly agree	disagree	agree
strongly agree	disagree	disagree	agree	agree	agree	agree
agree	disagree	disagree	disagree	agree	agree	disagree
strongly agree	strongly disagre	agree	disagree	strongly agree	agree	disagree
disagree	disagree	disagree	agree	disagree	disagree	agree
agree	strongly agree	agree	strongly agree	agree	agree	disagree
strongly disagre	agree	agree	strongly agree	disagree	disagree	strongly disagree
disagree	agree	disagree	disagree	agree	agree	strongly agree
agree	agree	strongly disagre	strongly disagre	agree	agree	disagree
agree	agree	disagree	agree	disagree	strongly agree	disagree
disagree	strongly agree	strongly agree	disagree	agree	agree	disagree
agree	strongly agree	strongly disagre	strongly agree	agree	agree	disagree
strongly agree	agree	agree	strongly agree	agree	strongly agree	disagree
strongly agree	disagree	disagree	agree	agree	agree	agree
strongly disagre	disagree	agree	disagree	disagree	agree	disagree
agree	disagree	disagree	strongly agree	disagree	agree	agree
disagree	agree	agree	agree	agree	disagree	strongly disagree
disagree	disagree	agree	agree	disagree	disagree	disagree
strongly agree	strongly agree	strongly disagre	strongly disagre	disagree	agree	strongly disagree
agree	strongly agree	agree	disagree	strongly agree	disagree	strongly agree

agree	strongly disagre	disagree	strongly agree	agree	strongly agree	strongly disagree
strongly agree	agree	strongly agree	disagree	agree	disagree	agree
strongly agree	agree	disagree	disagree	disagree	strongly agree	disagree
agree	agree	agree	agree	disagree	disagree	disagree
strongly agree	strongly agree	strongly agree	agree	strongly agree	disagree	strongly agree
strongly agree	strongly agree	disagree	agree	disagree	disagree	disagree
disagree	strongly disagre	disagree	strongly agree	disagree	agree	strongly disagree
disagree	agree	disagree	agree	agree	disagree	disagree
strongly agree	agree	disagree	disagree	agree	strongly disagre	disagree
agree	agree	strongly agree	disagree	agree	agree	disagree
agree	strongly agree	agree	disagree	strongly agree	strongly disagre	strongly disagree
agree	agree	disagree	strongly agree	agree	agree	disagree
strongly agree	disagree	strongly disagre	strongly agree	agree	disagree	disagree
agree	agree	agree	disagree	strongly agree	agree	agree
strongly agree	disagree	agree	agree	agree	agree	disagree
strongly disagre	disagree	disagree	strongly agree	disagree	disagree	disagree
disagree	strongly agree	agree	disagree	disagree	agree	strongly disagree
agree	disagree	agree	agree	disagree	agree	disagree
strongly agree	disagree	disagree	agree	strongly agree	agree	disagree
strongly agree	agree	agree	disagree	disagree	agree	disagree
agree	agree	strongly disagre	strongly disagre	disagree	disagree	strongly disagree
strongly agree	agree	strongly agree	agree	agree	agree	agree
disagree	strongly disagre	strongly disagre	strongly agree	disagree	strongly agree	disagree
agree	strongly agree	disagree	strongly agree	agree	agree	agree
agree	strongly agree	agree	disagree	disagree	disagree	disagree
strongly agree	strongly disagre	disagree	disagree	agree	agree	disagree
agree	agree	disagree	disagree	strongly agree	agree	strongly disagree
agree	disagree	agree	agree	strongly agree	agree	disagree
agree	disagree	strongly agree	agree	agree	disagree	disagree
disagree	agree	agree	strongly agree	strongly agree	disagree	disagree
agree	disagree	strongly agree	agree	agree	agree	disagree
agree	agree	agree	disagree	agree	agree	disagree
strongly agree	disagree	disagree	strongly disagre	disagree	strongly disagre	disagree
strongly agree	disagree	disagree	strongly agree	disagree	strongly agree	disagree
strongly agree	agree	disagree	strongly agree	agree	agree	strongly disagree

agree	strongly disagre	agree	strongly agree	disagree	agree	disagree
strongly agree	disagree	agree	agree	disagree	strongly agree	disagree
disagree	agree	agree	strongly disagre	agree	agree	strongly disagree
strongly agree	agree	strongly agree	strongly agree	agree	strongly agree	disagree
strongly agree	disagree	disagree	agree	agree	agree	agree
agree	agree	agree	disagree	disagree	strongly agree	strongly agree
disagree	agree	agree	strongly agree	strongly agree	agree	disagree
strongly agree	agree	disagree	agree	agree	strongly agree	agree
disagree	disagree	strongly agree	strongly agree	strongly agree	agree	agree
strongly agree	disagree	strongly agree	disagree	disagree	strongly agree	disagree
agree	disagree	disagree	disagree	disagree	agree	disagree
disagree	disagree	strongly agree	disagree	agree	agree	agree
disagree	agree	disagree	strongly agree	strongly agree	disagree	disagree
agree	agree	disagree	agree	agree	disagree	disagree
strongly disagre	strongly agree	agree	disagree	agree	agree	agree
strongly agree	agree	agree	strongly agree	disagree	strongly agree	strongly agree
strongly agree	agree	strongly agree	strongly agree	strongly agree	strongly agree	strongly disagree
disagree	disagree	disagree	agree	agree	agree	disagree
agree	strongly agree	disagree	disagree	agree	strongly agree	strongly agree
strongly agree	agree	strongly agree	agree	strongly agree	disagree	disagree
disagree	disagree	agree	agree	disagree	strongly agree	disagree
disagree	agree	disagree	disagree	strongly agree	agree	strongly disagree
disagree	strongly disagre	agree	strongly disagre	agree	agree	agree
strongly agree	strongly agree	disagree	disagree	agree	agree	disagree
agree	disagree	strongly agree	agree	agree	disagree	disagree
strongly agree	strongly disagre	disagree	agree	agree	disagree	agree
disagree	strongly agree	disagree	strongly agree	agree	agree	disagree
agree	strongly agree	agree	disagree	disagree	disagree	agree
agree	agree	agree	agree	agree	strongly agree	agree
agree	agree	agree	disagree	disagree	disagree	disagree
agree	disagree	strongly disagre	strongly disagre	disagree	agree	disagree
agree	agree	agree	disagree	agree	disagree	agree
agree	agree	agree	agree	agree	disagree	agree
strongly agree	disagree	agree	strongly agree	strongly agree	agree	disagree
agree	agree	agree	disagree	agree	agree	disagree

agree	agree	disagree	disagree	disagree	strongly agree	disagree
disagree	agree	disagree	agree	agree	strongly disagre	disagree
strongly agree	disagree	agree	strongly disagre	strongly agree	agree	strongly disagree
agree	agree	disagree	disagree	agree	agree	strongly disagree
strongly agree	disagree	strongly disagre	strongly agree	strongly agree	strongly disagre	agree
agree	disagree	disagree	agree	agree	agree	agree
strongly agree	agree	strongly agree	agree	agree	agree	disagree
strongly agree	strongly agree	disagree	disagree	agree	strongly agree	strongly disagree
agree	strongly agree	agree	disagree	strongly agree	disagree	disagree
strongly agree	agree	disagree	agree	disagree	strongly agree	disagree
agree	agree	agree	agree	agree	agree	strongly disagree
disagree	strongly agree	disagree	disagree	strongly agree	agree	disagree
agree	agree	agree	agree	strongly agree	disagree	strongly disagree
agree	disagree	agree	agree	disagree	disagree	agree
disagree	strongly agree	strongly agree	disagree	strongly agree	disagree	disagree
agree	disagree	strongly agree	strongly disagre	strongly agree	agree	disagree
strongly disagre	agree	agree	disagree	disagree	strongly agree	agree
agree	agree	agree	agree	agree	agree	strongly agree
disagree	agree	strongly agree	disagree	disagree	disagree	strongly disagree
agree	agree	disagree	agree	agree	disagree	disagree
disagree	agree	agree	strongly disagre	strongly agree	agree	disagree
agree	strongly agree	disagree	agree	disagree	strongly agree	strongly disagree
strongly agree	agree	agree	agree	agree	disagree	strongly disagree
strongly agree	agree	strongly agree	agree	disagree	disagree	agree
agree	agree	disagree	disagree	agree	disagree	disagree
strongly agree	disagree	disagree	agree	disagree	disagree	agree
agree	disagree	disagree	agree	disagree	agree	strongly disagree
strongly agree	disagree	agree	strongly disagre	strongly agree	disagree	disagree
disagree	disagree	agree	disagree	disagree	agree	agree
agree	disagree	disagree	disagree	strongly agree	disagree	disagree
agree	strongly agree	agree	disagree	disagree	disagree	strongly disagree
strongly disagre	agree	agree	strongly agree	strongly agree	disagree	strongly disagree
agree	disagree	disagree	disagree	strongly agree	agree	agree
strongly agree	strongly agree	strongly agree	disagree	strongly agree	agree	strongly disagree
disagree	strongly disagre	disagree	agree	strongly agree	disagree	strongly agree

agree	strongly agree	disagree	strongly agree	strongly agree	strongly agree	strongly disagree
disagree	strongly disagre	disagree	strongly agree	strongly agree	agree	strongly agree
strongly agree	strongly agree	disagree	disagree	disagree	strongly disagre	disagree
agree	disagree	strongly disagre	agree	disagree	agree	agree
agree	strongly agree	disagree	agree	agree	disagree	strongly agree
agree	disagree	strongly agree	disagree	agree	disagree	agree
disagree	agree	agree	disagree	strongly agree	agree	strongly agree
agree	strongly agree	disagree	agree	disagree	strongly agree	disagree
strongly agree	disagree	agree	strongly agree	disagree	disagree	disagree
strongly agree	strongly agree	agree	strongly agree	agree	disagree	disagree
disagree	disagree	disagree	disagree	strongly agree	agree	disagree
strongly agree	agree	strongly agree	agree	disagree	strongly agree	agree
disagree	disagree	disagree	agree	strongly agree	disagree	disagree
strongly agree	strongly disagre	agree	disagree	strongly agree	strongly agree	disagree
agree	strongly agree	disagree	agree	agree	strongly disagre	strongly agree
strongly agree	strongly agree	agree	disagree	disagree	disagree	agree
strongly agree	agree	disagree	disagree	disagree	disagree	strongly disagree
disagree	strongly agree	strongly agree	disagree	disagree	strongly agree	disagree
strongly agree	disagree	strongly agree	disagree	agree	disagree	disagree
strongly agree	strongly agree	agree	agree	strongly agree	agree	agree
strongly agree	disagree	disagree	disagree	agree	disagree	disagree
strongly agree	disagree	agree	strongly agree	disagree	strongly agree	agree
agree	agree	disagree	strongly disagre	disagree	disagree	strongly disagree
strongly agree	disagree	strongly disagre	disagree	agree	strongly agree	agree
agree	agree	strongly disagre	agree	disagree	agree	disagree
strongly agree	disagree	disagree	strongly agree	disagree	agree	strongly disagree
agree	agree	strongly agree	agree	strongly agree	disagree	disagree
agree	agree	strongly agree	agree	disagree	strongly agree	strongly agree
agree	agree	agree	strongly agree	agree	disagree	agree
disagree	disagree	agree	strongly agree	disagree	strongly agree	strongly agree
strongly agree	agree	disagree	disagree	agree	strongly agree	disagree
strongly agree	disagree	agree	disagree	disagree	agree	disagree
agree	disagree	agree	agree	disagree	strongly agree	disagree
agree	agree	disagree	agree	disagree	agree	agree
strongly agree	agree	agree	disagree	agree	agree	disagree

agree	strongly agree	disagree	strongly agree	strongly agree	disagree	disagree
strongly disagre	disagree	disagree	agree	disagree	strongly agree	strongly disagree
agree	disagree	disagree	agree	agree	disagree	disagree
agree	agree	disagree	disagree	agree	agree	strongly agree
strongly agree	disagree	agree	agree	disagree	strongly agree	strongly agree
strongly agree	agree	disagree	agree	strongly agree	disagree	strongly disagree
agree	disagree	agree	agree	agree	disagree	strongly disagree
strongly disagre	agree	agree	disagree	agree	strongly agree	disagree
strongly agree	disagree	agree	agree	agree	strongly disagre	disagree
disagree	disagree	strongly agree	agree	disagree	disagree	strongly disagree
agree	disagree	agree	agree	agree	agree	agree
strongly agree	agree	agree	agree	disagree	agree	disagree
agree	strongly agree	disagree	disagree	agree	disagree	strongly disagree
agree	disagree	strongly agree	agree	disagree	agree	agree
agree	disagree	disagree	agree	agree	agree	disagree
agree	agree	disagree	agree	strongly agree	disagree	disagree
agree	strongly agree	strongly agree	strongly agree	disagree	disagree	strongly disagree
agree	agree	strongly disagre	disagree	agree	strongly disagre	agree
disagree	disagree	disagree	strongly agree	strongly agree	agree	agree
agree	disagree	disagree	disagree	disagree	agree	disagree
strongly agree	strongly agree	strongly agree	agree	disagree	disagree	agree
agree	agree	disagree	agree	agree	agree	strongly agree
disagree	agree	disagree	disagree	disagree	disagree	agree
agree	agree	agree	strongly disagre	disagree	disagree	agree
disagree	agree	agree	agree	strongly agree	agree	disagree
strongly agree	disagree	strongly agree	agree	agree	strongly disagre	disagree
agree	agree	disagree	disagree	agree	disagree	agree
strongly agree	strongly agree	agree	strongly agree	disagree	agree	agree
strongly agree	agree	agree	strongly agree	disagree	disagree	strongly agree
agree	strongly agree	strongly agree	agree	agree	agree	strongly agree
strongly agree	strongly agree	disagree	agree	disagree	agree	disagree
strongly agree	agree	agree	strongly agree	agree	agree	strongly disagree
strongly agree	disagree	strongly agree	strongly agree	disagree	agree	disagree
strongly agree	agree	disagree	agree	agree	strongly agree	disagree
strongly agree	strongly disagre	agree	agree	disagree	strongly agree	disagree

disagree	disagree	agree	agree	strongly agree	agree	agree
agree	disagree	strongly disagre	disagree	disagree	agree	agree
strongly agree	disagree	agree	agree	disagree	agree	disagree
strongly agree	agree	strongly agree	agree	disagree	strongly agree	agree
strongly agree	strongly disagre	agree	disagree	strongly agree	agree	strongly disagree
disagree	agree	disagree	disagree	strongly agree	agree	disagree
agree	strongly agree	disagree	disagree	agree	strongly agree	strongly disagree
disagree	strongly agree	agree	strongly agree	strongly agree	disagree	disagree
agree	agree	disagree	disagree	disagree	agree	disagree
strongly agree	disagree	disagree	strongly agree	strongly agree	strongly agree	agree
agree	strongly agree	agree	agree	strongly agree	agree	agree
disagree	agree	strongly agree	disagree	strongly agree	strongly agree	agree
disagree	disagree	strongly agree	strongly agree	disagree	disagree	agree
strongly agree	agree	strongly agree	strongly disagre	strongly agree	disagree	disagree
disagree	strongly agree	disagree	agree	agree	strongly agree	strongly disagree
disagree	strongly agree	agree	disagree	agree	agree	agree
strongly agree	disagree	agree	strongly disagre	disagree	strongly agree	agree
strongly agree	agree	agree	agree	agree	disagree	disagree
agree	agree	agree	disagree	disagree	disagree	disagree
agree	agree	strongly agree	strongly agree	strongly agree	strongly agree	strongly disagree
strongly agree	agree	disagree	strongly agree	strongly agree	agree	disagree
strongly disagre	strongly agree	strongly agree	agree	disagree	strongly agree	disagree
agree	strongly agree	agree	strongly agree	strongly agree	disagree	disagree
disagree	agree	disagree	disagree	disagree	agree	disagree
strongly agree	agree	strongly agree	disagree	disagree	strongly agree	disagree
strongly agree	agree	strongly agree	disagree	agree	agree	strongly disagree
disagree	agree	agree	agree	strongly agree	agree	strongly agree
strongly agree	strongly agree	strongly agree	disagree	strongly agree	agree	disagree
agree	disagree	disagree	strongly agree	strongly agree	disagree	disagree
disagree	disagree	agree	agree	disagree	disagree	agree
agree	strongly agree	disagree	strongly agree	agree	disagree	agree
strongly agree	disagree	strongly agree	agree	disagree	agree	disagree
