# Supplementary material for: Engagement of primary care physicians in medication decision-making for patients with multimorbidity in China: A cross-sectional study
Source: PLoS One. 2026 Mar 26;21(3):e0344518. doi: 10.1371/journal.pone.0344518 (PMC13020813; doi:10.1371/journal.pone.0344518)
Supplement: Attachment — Submitted filename: Response to Reviewers 2.2.docx [file pone.0344518.s004.docx]

Dear editor and reviewers:

Thank you for your letter and for the reviewers’ comments concerning our manuscript entitled “Engagement of Primary Care Physicians in Medication Decision-making for Patients with Multimorbidity in China: A Cross-sectional Study (ID: PONE-D-25-67618)”. The comments were insightful and helpful for improving our manuscript. We appreciate the time and effort invested in reviewing our work, and we believe that the feedback has significantly improved the quality of our research. Therefore, we have carefully revised our manuscript according to the comments. Revisions in the text are shown using yellow highlighting for additions, and strike through font for deletions. The point-by-point responses to the reviewer’s comments are listed below:

**Responses to the journal requirement**:

**【COMMENT 1】**Please ensure that your manuscript meets PLoS ONE’s style requirements, including those for file naming.

**【RESPONSE 1】** Thank you very much for your thoughtful review and for providing clear guidance regarding the manuscript formatting. I have carefully revised the manuscript to ensure it fully complies with PLoS ONE’s style requirements, including all adjustments related to formatting, structure, and file naming as specified. I sincerely appreciate your time and effort in reviewing our work, and I remain fully open to any further suggestions or adjustments that may be needed.

**【COMMENT 2 AND 3】**All PLOS journals now require all data underlying the findings described in their manuscript to be freely available to other researchers, either 1. In a public repository, 2. Within the manuscript itself, or 3. Uploaded as supplementary information.

**【RESPONSE 2 AND 3】** Thank you for highlighting the important data availability policy of PLOS journals. We fully support the principle of open data. In accordance with this requirement, we have now deposited the fully anonymized raw questionnaire data underlying the findings of this study in the public repository Zenodo. The dataset is freely accessible under the following DOI: 10.5281/zenodo.18577776.

We have also updated the Data Availability Statement within the manuscript to explicitly cite this DOI and facilitate access for all interested researchers. We believe this ensures full transparency and supports the reproducibility of our work.

(Results－Respondent Characteristics and Work-related Information)

The electronic questionnaires were distributed to 386 PCPs, and 363 PCPs (response rate: 94%) responded and agreed to participate in the study. However, 17 questionnaires were excluded due to invalid responses, leaving 346 valid questionnaires for data analysis. The specific questionnaire data can be found in S2 File.

(Data available statement)

**Data Available Statement**

~~Due to privacy and confidentiality restrictions under Chinese regulations and the conditions of participant consent, the full dataset cannot be made publicly available. De-identified data can be provided to qualified researchers upon reasonable request, subject to a formal data use agreement. Requests should be directed to the corresponding author.~~ All relevant data are within the manuscript and its Supporting Information files (S2 File).

(Supporting information)

**S2 File. PCP_MedDecision_Multimorbidity_Survey_Data.** The original data and statistics of the questionnaire survey on Engagement of Primary Care Physicians in Medication Decision-making for Patients with Multimorbidity in China: A Cross-sectional Study

**【COMMENT 4】** Your ethics statement should only appear in the Methods section of your manuscript. If your ethics statement is written in any section besides the Methods, please delete it from any other section.

**【RESPONSE 4】**Thank you for your clear guidance regarding the placement of the ethics statement. In accordance with your instructions, I have reviewed the manuscript and ensured that the ethics statement now appears exclusively in the Methods section. The statement previously included in the latter part of the manuscript has been removed.

**【COMMENT 5】**Please include captions for your Supporting Information files at the end of your manuscript, and update any in-text citations to match accordingly.

**【RESPONSE 5】**Thank you for your valuable comment. We have carefully revised the manuscript according to the journal’s formatting requirements. Captions for the Supporting Information files have been added at the end of the manuscript, and all relevant in-text citations have been updated accordingly. The description of the custom questionnaire, previously noted in the Methods section, is now consistently referenced both within the text and in the newly added Supporting Information captions.

(Methods－Instrument) The questionnaire used in this study was self-developed ~~(see supplementary file)~~. The questionnaire used in the survey can be found in S1 File. The initial item pool was generated based on a comprehensive literature review of multimorbidity management, medication review, deprescribing, and clinical decision-making frameworks [1–6, 20–22].

(Supporting information) **S1 File. A survey on primary care physicians’ engagement in medication decision-making for patients with multimorbidity.**

**【COMMENT 6】**If the reviewer comments include a recommendation to cite specific previously published works, please review and evaluate these publications to determine whether they are relevant and should be cited.

**【RESPONSE 6】**Thank you for your kind reminder. We have carefully evaluated each recommended publication. We have selectively incorporated citations only where the referenced work provides direct and substantive support for the methodological framework or contextual discussion within our manuscript.

**Responses to the reviewer 1**

**【COMMENT 1】**Good description of PHC in China. What is the role of specialist physicians regarding medication prescribing? In our research we found that PCPs feel very uncomfortable adjusting therapies or deprescribing medications that they did not themselves prescribe. How does the PCP-specialist physician relationship impact medication management?

**【RESPONSE 1】**Thank you for raising this important point regarding the relationship between PCPs and specialists in medication management. We have revised the Introduction to explicitly address this dynamic, noting that clinical inertia often stems from PCPs’ hesitancy to adjust prescriptions initiated by specialists. This addition further clarifies a key contextual barrier in our study setting.

(Introduction) Numerous barriers hinder their ability to optimize treatment, including clinical inertia, patient resistance to changes in medication regimens, limited access to decision support tools, inadequate training in managing polypharmacy, and time constraints during consultations [12,13]. Specifically, clinical inertia among PCPs often stems from their hesitancy to modify specialist-initiated regimens due to deference to authority, uncertainty about prescribing rationale, and legal concerns, thus compromising medication safety and care quality [14]. Moreover, the lack of clinical guidelines and insufficient evidence for treating patients with multimorbidity make it difficult to formulate individualized medication plans [15].

**References added:**

[14] Phillips LS, Branch WT, Cook CB, Doyle JP, El-Kebbi IM, Gallina DL, et al. Clinical inertia. Ann Intern Med. 2001; 135(9):825-34. doi: 10.7326/0003-4819-135-9-200111060-00012.

**【COMMENT 2】**Thank you for providing the survey. Again, lack of inclusion about items related to adjusting therapies that the provider itself did not start is a concern since the authors conducted a literature review.

**【RESPONSE 2】**Thank you for raising this important point regarding the scope of our survey. We wish to clarify that the questionnaire items were designed to assess PCPs’ decision-making engagement in a broad clinical context, specifically, when initiating a new prescription or modifying an existing medication regimen for a patient, irrespective of whether the original therapy was started by the PCP or another provider (e.g., a specialist). Thus, the instrument captures participatory decision-making practices across both self-initiated and externally initiated therapies under the PCP’s ongoing management.

**【COMMENT 3】**Manuscript line 101: Please cite a reference for the final statement in this paragraph.

**【RESPONSE 3】**Thank you for your suggestion regarding the citation for the psychometric properties. We would like to clarify that the reported metrics (Cronbach’sα, ICC, and CFI) were derived from the pilot testing phase of this study, which was conducted to assess the psychometric properties of the self-developed questionnaire. This preliminary validation confirms the instrument's robustness for the main survey. In line with your advice, we have supplemented the relevant methodological literature on psychometric evaluation in this section.

(Methods－Instrument) The instrument demonstrated good internal consistency (Cronbach’s α= 0.87 for the full scale), strong test–retest reliability (intraclass correlation coefficient = 0.84 over a two-week interval) and satisfactory construct validity (χ²/df=1.98, CFI=0.93, TLI=0.91, RMSEA=0.06) [18].

**References:**

[18] Kline RB. Principles and practice of structural equation modeling, 3rd ed. New York: Guilford Publications, 2011.

**【COMMENT 4】**Why was NEVER not included in the likert type scale since ALWAYS is included.

**【RESPONSE 4】**We sincerely appreciate the reviewer’s insightful question regarding the response options in our questionnaire. Our decision to use a three-point scale ("always," "occasionally," "rarely") was based on methodological and practical considerations. We opted against including a "never" option to minimize potential social desirability bias and respondent discomfort, as an absolute term might be perceived as an admission of not performing a core professional activity. The term “rarely” was chosen to capture low-frequency behaviors while maintaining a constructive and less prescriptive tone. Furthermore, from an analytical perspective, we anticipated that a “never” response would be selected infrequently, potentially creating a highly skewed distribution that could complicate subsequent statistical analyses. The chosen scale provided a balanced distribution suitable for our analysis while maintaining a constructive response tone.

**【COMMENT 5】**Please provide n’s where you have percentages.

**【RESPONSE 5】**Thank you for this helpful suggestion. We have revised the manuscript to ensure that all percentages reported in the Results section are now accompanied by their corresponding absolute numbers (n). As the changes are made throughout the relevant sections, we have not listed them individually here but have implemented them comprehensively in the revised manuscript to enhance the clarity and transparency of our data presentation.

(Results－Respondent Characteristics and Work-related Information)

...

Regarding work-related characteristics, 63.9% (n=221) of PCPs had completed standardized residency training, and 71.7% (n=248) were responsible for family doctor contract services. Two-thirds (n=233, 67.3%) practiced in rural PHC settings. Approximately a quarter reported a daily outpatient volume exceeding 50 patients. In terms of institutional support, 64.5% (n=223) had participated in pharmaceutical training in the past year, 75.4% (n=261) reported routine prescription reviews in their facilities, and 82.7% (n=286) indicated access to electronic drug information systems. When facing challenges in medication decision-making, 42.2% of PCPs (n=146) reported collaborating with pharmacists.

...

**【COMMENT 6 AND 7】**Revise from “...nearly half...” to the majority. Revise from "...junior or intermediate professional titles..." to were resident physicians or attending physicians.

**【RESPONSE 6 AND 7】**Thank you for your kind reminder. We have carefully revised the wording to ensure accuracy and clarity in the manuscript. The text has been updated as suggested, specifically changing “nearly half” to “the majority” and revising “held junior or intermediate professional titles” to “were resident physicians or attending physicians”.

(Result－Respondent Characteristics and Work-related Information) Among the respondents, ~~nearly half~~ the majority (n=180, 52.0%) were male, with a mean age of 37.8 years (range: 24–59). The majority of participants had fewer than 15 years of practice experience (n=217, 62.7%), ~~held junior or intermediate professional titles,~~ with most identifying as resident physicians or attending physicians (n=272, 78.7%), and had obtained a bachelor’s degree (n=284, 82.1%) (Table 1).

**【COMMENT 8】**Would be helpful to know how representative the sample is to the Chinese PCP population. Please revise the Methods to include some statistical comparisons and present the findings here.

**【RESPONSE 8】**Thank you for this valuable suggestion regarding the representativeness of our sample. In response, we have added a concise comparative statement in the Results section (following the presentation of participants’ demographic and practice characteristics). By referencing the latest national health statistics (e.g., China Health Statistics Yearbook 2024), we note that the gender, age, educational, and professional title distribution of our sample aligns closely with the documented profile of physicians in China—where, for instance, 52.1% are male, 35.2% are aged 35–44, and 78.4% hold a bachelor’s degree or higher. This addition references national statistical data and indicates that the profile of our sample aligns broadly with that of the primary care physician population in China, thereby supporting the reasonable representativeness of our cohort within the studied context.

(Result－Respondent Characteristics and Work-related Information) The majority of participants had fewer than 15 years of practice experience (n=217, 62.7%), with most identifying as resident physicians or attending physicians (n=272, 78.7%), and had obtained a bachelor’s degree (n=284, 82.1%) (Table 1). The demographic profile of our sample was largely consistent with the distribution patterns reported for primary care physicians in national health statistics, supporting its representativeness to a certain extent [27].

**References added:**

[27] National Health Commission of China. 2024 China Health Statistics Yearbook. Beijing: Peking Union Medical College Press, 2025.

**【COMMENT 9】**Is it common for PCPs not to have completed any residency training? Also, is it possible that there might be some physicians who were pharmacists or had some specialized pharmacology training prior to becoming a PCP?

**【RESPONSE 9】**Thank you for your questions regarding the training background of physicians in China. We would like to provide a detailed explanation regarding this matter. Approximately a decade ago, China introduced a three-year standardized residency training program, which is now generally required for medical graduates to obtain a training completion certificate before practicing independently. Regarding pharmacology training, all PCPs in China are graduates of clinical medicine programs, while pharmacists cannot transition to become physicians. The pharmacology education these physicians receive during both undergraduate study and residency training is fragmented and limited, lacking the depth of specialized pharmacy training.

**【COMMENT 10】**Please provide descriptive statistics for the “score” so readers can better understand where the PCPs landed in terms of medication practices.

**【RESPONSE 10】**Thank you for this valuable comment. We agree that descriptive statistics regarding the overall engagement scores would help readers better interpret the level of PCPs' involvement in medication decision-making. We acknowledge that this information was initially missing from the manuscript.

In response to your suggestion, we have now added a paragraph under Results section (PCPs’ Engagement of Medication Decision-making for Patients with Multimorbidity) to present the descriptive statistics of the engagement scores. We believe this addition provides a clearer and more comprehensive overview of PCPs’ medication decision-making practices.

(Results－PCPs’ Engagement of Medication Decision-making for Patients with Multimorbidity) Based on the 20-item questionnaire (total possible score: 40), the 346 PCPs had a mean engagement score of 31.8 (ranging from 21 to 38). The majority (n=227, 65.6%) scored between 31 and 35 points. All participants scored above half of the total possible score.

Before initiating treatment plans, 72.5% of PCPs (n=251) reported “always” reviewing patients’ medical and medication histories, while 68.2% (n=236) explored the purpose of the visit, and 70.2% (n=243) addressed patients’ prioritized needs.

**【COMMENT 11】**The sentence “...align with barriers previously reported in both...” needs to be finished.

**【RESPONSE 11】**Thank you for your kind reminder. We have carefully revised the wording to ensure the completeness and clarity of the sentence. The incomplete statement in the original text has been corrected by adding the missing word, specifically amending “resource-constrained” to “resource-constrained countries”.

(Discussion) The work-related factors identified in our study, such as limited access to decision-support resources, insufficient training in polypharmacy management, and time pressures during consultations, align with barriers previously reported in both high-income and resource-constrained countries [12,13,29,30].

**【COMMENT 12】**The sentence “...currently clinical guidelines which strongly advocate...” needs to be cited. Preferably one about polypharmacy or multimorbidity management.

**【RESPONSE 12】**Thank you for your thoughtful comment regarding the need to substantiate the statement about clinical guidelines. We acknowledge that the citation in our initial submission could have been more explicit. Upon review, we confirm that the recommended reference on polypharmacy and multimorbidity management (Muth et al., 2019) was already included in our manuscript’s reference list. To strengthen the support for this specific claim, we have now explicitly cited this guideline at the relevant point in the sentence.

(Discussion) This stands in contrast to current clinical guidelines, which strongly advocate for patient-centered care and shared decision-making, particularly in the management of multimorbidity [31].

**References:**

[31] Muth C, Blom JW, Smith SM, Johnell K, Gonzalez-Gonzalez AI, Nguyen TS, et al. Evidence supporting the best clinical management of patients with multimorbidity and polypharmacy: a systematic guideline review and expert consensus. J Intern Med. 2019, 285(3):272-288. doi: 10.1111/joim.12842.

**【COMMENT 13】**The sentence "...research has demonstrated that engaging patients in decision-making processes...", has research found SDM improves medication adherence and/or disease outcomes?

**【RESPONSE 13】**Thank you for your valuable suggestion regarding the need to substantiate the link between SDM and patient outcomes. While our originally cited reference (Laviana et al., 2017) does state that involved patients “have better outcomes”, we acknowledge that this correlational language does not constitute direct, quantified empirical data on specific clinical endpoints. To more accurately reflect the available evidence and enhance the academic rigor of our argument, we have revised the relevant sentence. We have changed the phrasing from “...leads to better clinical outcomes...” to the more measured “...may have a positive association with clinical outcomes...” This modification aims to precisely convey the supportive yet indicative relationship suggested by the literature, rather than asserting a definitive causal link. We appreciate your guidance in prompting this clarification and are open to any further suggestions.

(Discussion) Evidence from international studies has shown that shared decision-making not only improves medication adherence and patient satisfaction but also ~~leads to better clinical outcomes in multimorbid populations~~ may have a positive association with clinical outcomes in multimorbid populations [31].

**【COMMENT 14】**The sentence “...aligns with a well-documented limitation in current practice:...” needs to be cited.

**【RESPONSE 14】**Thank you for your thoughtful comment regarding the need to substantiate the statement about the well-documented limitation in current practice. We acknowledge that the citation in our initial submission could have been more explicit. Upon review, we confirm that the recommended reference (Wang et al., Ageing Res Rev, 2024), which systematically reviews the limitations of current disease-specific guidelines in addressing multimorbidity, was already included in our manuscript's reference list. To directly address your point and strengthen the support for this specific claim, we have now explicitly cited this review at the relevant point in the sentence.

(Discussion) This finding aligns with a well-documented limitation in current practice: most clinical guidelines remain disease-specific and fail to account for the complexity of managing patients with multiple coexisting conditions [34].

**References:**

[34] Wang Z, Zhu D, Zhang H, Wang L, He H, Li Z, et al. Recommendations and quality of multimorbidity guidelines: A systematic review. Ageing Res Rev. 2024; 102:102559. DOI: 10.1016/j.arr.2024.102559.

**Responses to the reviewer 2**

**【COMMENT 1】**The first cutoff point reported for the “number of outpatient visits per day” is high (≤50 patients per day). This seems quite striking. Considering that the question was free text (open question), I suggest exploring others range, or explaining how and why this cutoff was defined.

**【RESPONSE 1】**Thank you for your insightful comment and for raising this important point regarding the cutoff values for the daily number of outpatient visits. We appreciate your careful review. In our survey, which used an open-ended question, the reported daily patient volumes varied considerably, ranging from single digits to over 150 visits per day. Given this wide dispersion and the substantial gap between the minimum and maximum values, we aimed to create categories that encompassed the entire range while remaining practical for statistical analysis.

The selected cutoffs (≤50; 51–100; 101–150; ≥151) were defined to capture the full spectrum of reported daily visits. It ranged from single digits to over 150 within distinct workload categories, while avoiding excessive stratification that could create too many groups with small sample sizes and compromise the clarity and statistical power of the analysis.

**【COMMENT 2】**Please revise the statement “one-third reported a daily outpatient volume exceeding 50 patients” (line 186) to “a quarter reported…”, as this corresponds to approximately 25%.

**【RESPONSE 2】**Thank you for your kind reminder. We have carefully revised the wording to ensure consistency in data presentation throughout. The incorrect statistical data in the original text has been corrected, specifically changing “one third” to “one quarter”.

(Results－Respondent Characteristics and Work-related Information) Two-thirds (n=233, 67.3%) practiced in rural PHC settings. Approximately ~~one-third~~ a quarter reported a daily outpatient volume exceeding 50 patients. In terms of institutional support, 64.5% (n=223) had participated in pharmaceutical training in the past year, 75.4% (n=261) reported routine prescription reviews in their facilities, and 82.7% (n=286) indicated access to electronic drug information systems.

**【COMMENT 3】**The proportion of resident physicians represents almost half (46%) of all respondents. This is a significative characteristic of the sample, and its impact on the results should be discussed.

**【RESPONSE 3】**Thank you for raising this important observation regarding the composition of our survey respondents. We would like to clarify that within the Chinese healthcare system, this term refers specifically to a professional title rank for clinicians who have not yet attained intermediate title, rather than denoting postgraduate residency trainees as commonly understood in some other systems. Furthermore, professional promotion for doctors in China is challenging, particularly among PCPs, leading many experienced clinicians to remain within this title category. This characteristic may influence the interpretation of our results, and we have therefore incorporated a dedicated discussion of this potential limitation in the revised manuscript.

(Discussion) Nevertheless, we included participants from both urban and rural (PHC facilities to enhance representativeness and capture a range of practice environments. Furthermore, the high proportion of resident physicians (46%) in our sample, who typically have less experience and lower involvement in complex decision-making, may limit the generalizability of the findings to the broader population. ~~Second~~ Third, some respondents may not have fully expressed their perspectives due to time constraints, recall bias, or cognitive limitations, which could have influenced the accuracy of the self-reported data. ~~Third~~ Besides, there is a possibility of social desirability bias, as the survey was administered by local health authorities.

**【COMMENT 4】**Regarding the highest academic degree, it is not clear in the manuscript what is meant by “college” and “bachelor” within the context of Chinese medical education and training, especially given international differences in educational systems.

**【RESPONSE 4】**Thank you for this important clarification request. The international differences in the education system that you pointed out are indeed an area where our initial writing did not fully address. We have provided a clearer explanation for this academic degree in the revised version.

In the context of Chinese medical education, “college” refers to a sub-bachelor’s qualification (typically a 3-year diploma or associate degree), while “bachelor” denotes a full undergraduate degree (usually a 4- or 5-year program conferring a bachelor's degree). To avoid confusion, we will revise the manuscript to explicitly define these terms in the Methods section and, most importantly, change the “College” category label to “Below the bachelor” in all relevant text.

(Results－Respondent Characteristics and Work-related Information－Table 1)

| Education background | ~~College~~ Below the bachelor | 59 (17.1) |
| --- | --- | --- |
|  | Bachelor | 284 (82.1) |
|  | Master or doctor | 3 (0.9) |

We greatly appreciate the reviewers’ and editors’ valuable time and insightful comments on our manuscript. These suggestions have been very helpful in strengthening our work. We have carefully revised the manuscript accordingly and welcome any further feedback for improvement. We look forward to your consideration and remain ready to make additional revisions as needed. Thank you again for your thoughtful review.

Corresponding Author: Zhijie Xu, MD

Email: zhijiexu@zju.edu.cn (ZX)
